# Michael addition-based probes for ratiometric fluorescence imaging of protein *S*-depalmitoylases in live cells and tissues†
†Electronic supplementary information (ESI) available: Materials and methods, synthetic procedures, supplementary figures and data. See DOI: 10.1039/c7sc02805a


**DOI:** 10.1039/c7sc02805a

**Published:** 2017-09-11

**Authors:** Michael W. Beck, Rahul S. Kathayat, Candace M. Cham, Eugene B. Chang, Bryan C. Dickinson

**Affiliations:** a Department of Chemistry , The University of Chicago , 5801 South Ellis Avenue , Chicago , Illinois 60637 , USA . Email: Dickinson@UChicago.edu; b Department of Medicine , The University of Chicago , 5801 South Ellis Avenue , Chicago , Illinois 60637 , USA

## Abstract

The reversible modification of cysteine residues through thioester formation with palmitate (protein *S*-palmitoylation) is a prevalent chemical modification that regulates the function, localization, and stability of many proteins. Current methods for monitoring the “erasers” of *S*-palmitoylation, acyl-protein thioesterases (APTs), rely on destructive proteomic methods or “turn-on” probes, precluding deployment in heterogeneous samples such as primary tissues. To address these challenges, we present the design, synthesis, and biological evaluation of Ratiometric Depalmitoylation Probes (RDPs). RDPs respond to APTs with a robust ratiometric change in fluorescent signal both *in vitro* and in live cells. Moreover, RDPs can monitor endogenous APT activities in heterogeneous primary human tissues such as colon organoids, presaging the utility of these molecules in uncovering novel roles for APTs in metabolic regulation.

## Abstract 2

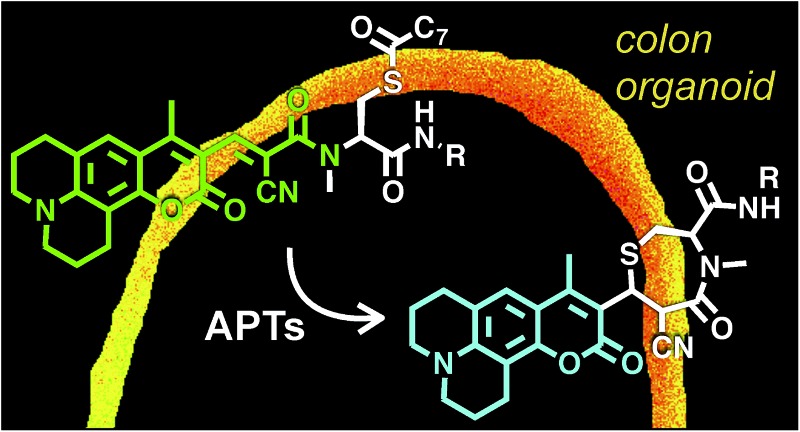
Ratiometric fluorescent probes for cysteine palmitoylation “erasers” permit live cell and tissue imaging of endogenous enzyme activities.

## Introduction

Protein *S*-palmitoylation is the modification of cysteine residues through thioester formation with palmitate, a prevalent cellular lipid.^1^,^2^ Estimates suggest that up to 10% of the human proteome is susceptible to regulation through *S*-palmitoylation,^3^ including oncogenic proteins such as Ras and BAX, and key proteins for neural activity such as PSD-95.^4^–^8^ The *S*-palmitoylation status of a given target protein is controlled through the balance of acyl-transferases that install the lipid, and protein depalmitoylase “eraser” proteins that remove the modification.^9^ In humans, there are two well-known cytosolic *S*-palmitoylation erasers, Acyl-Protein Thioesterase 1 and 2 (APT1 and APT2), and several recently-identified erasers, including the ABHD17 family of metabolic serine hydrolase proteins.^10^,^11^ There is growing evidence that the *S*-palmitoylation state of the proteome is dynamic and changes in response to a variety of physiological processes such as growth factor signaling, analogous to protein phosphorylation.^6^,^12^–^18^ Advances in proteomic and chemical biology technologies allow proteome-wide mapping of *S*-palmitoylated proteins,^9^,^19^ but deconvoluting the role of dynamic alterations to the *S*-depalmitoylases remains challenging. Genetic or pharmacological perturbation of the *S*-depalmitoylases can lead to unpredictable compensatory mechanisms, because only a handful of APTs govern the regulation of thousands of individual sites in the proteome. Therefore, methods to directly measure the activity levels of the *S*-palmitoylation erasers in live cells are critically needed to probe the effects and mechanism of this dynamic lipid chemical modification.

Live-cell interrogation of protein *S*-palmitoylation dynamics has been achieved by technically-cumbersome or indirect measurements such as monitoring the trafficking of microinjected fluorescent protein substrates or cell-permeable substrate mimetics.^17^,^20^,^21^ Recently, we unveiled Depalmitoylation Probes (DPPs), small molecule fluorescent probes that utilize a carbamate cleavage mechanism to release a pro-fluorophore upon thioesterase activity.^15^ DPPs are capable of monitoring the *S*-palmitoylation eraser proteins in live cells due to the large signal turn-on upon reaction with the target eraser protein. However, deploying turn-on probes such as the DPPs in heterogeneous samples is challenging, because unequal probe uptake and distribution can cause both false positives and false negatives. The dynamic regulation of the APTs during metabolic signaling in primary samples and complex tissues is of substantial interest due to the connections between *S*-palmitoylation and lipid homeostasis.^6^,^22^ As such, we sought to develop ratiometric fluorescent probes for *S*-palmitoylation eraser proteins that permit normalization of uptake. We now report the design, synthesis, and application of Ratiometric Depalmitoylation Probes (RDPs), a general ratiometric platform for detecting cysteine PTM eraser proteins that uses a Michael reaction mechanism. RDPs respond to APTs with a robust ratiometric response both *in vitro* and in live cells, and can be deployed to monitor endogenous *S*-depalmitoylation eraser activity levels in primary human colon organoids and in cell culture models of lipid stress.

## Results and discussion

### Design and synthesis of RDPs

**Scheme 1:**
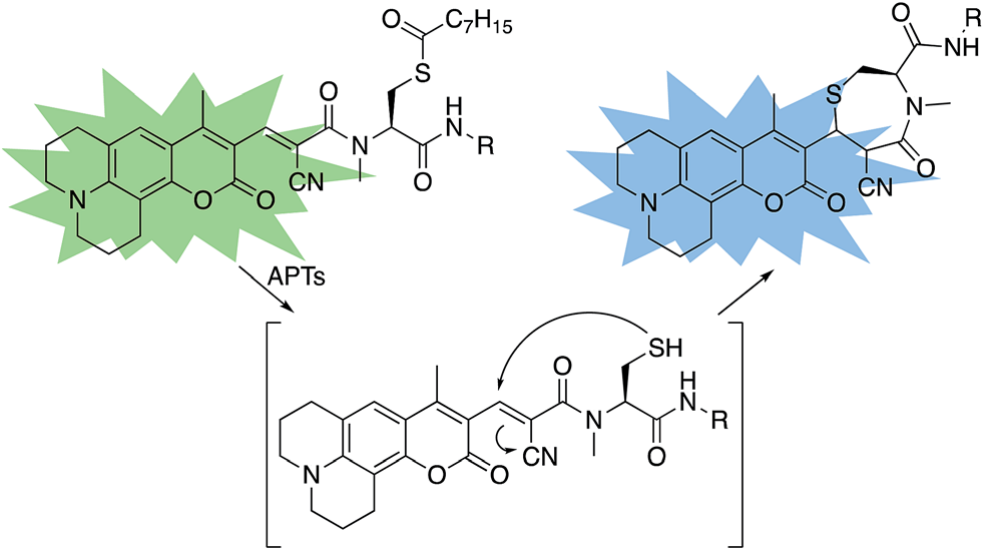
Design of RDPs. A peptide-based *S*-acylated APT substrate is appended to an aminocoumarin fluorophore. APT activity on the substrate results in a Michael reaction with a cyanoalkene linker, resulting in a blueshift in the probe.

**Scheme 2:**
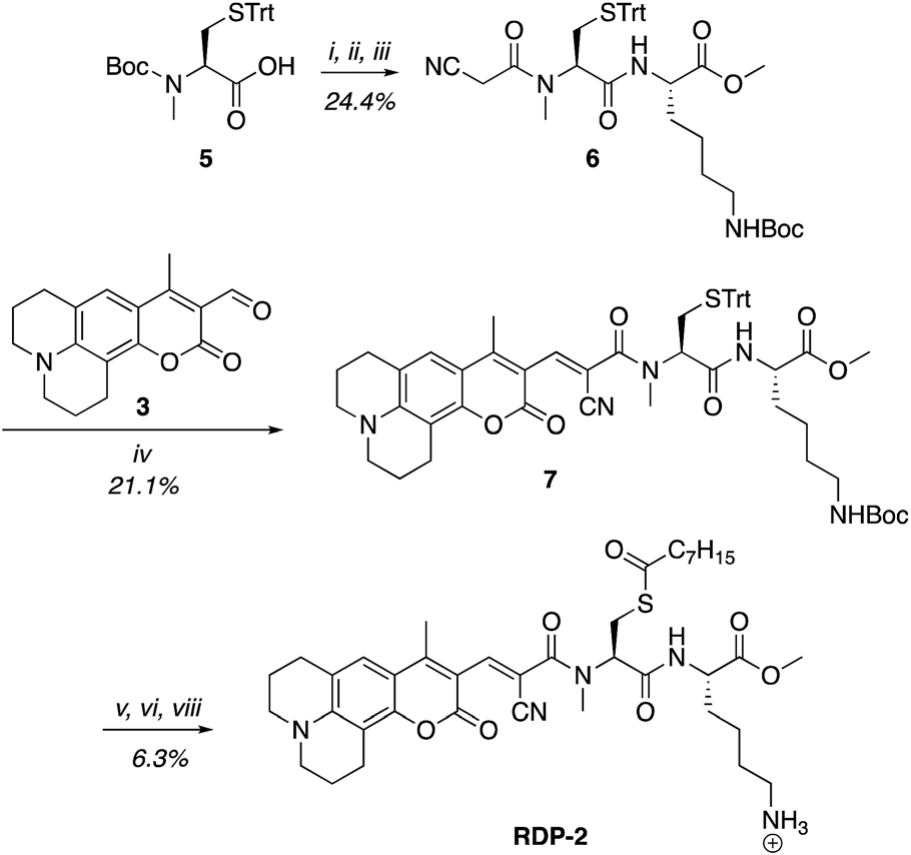
Synthesis of Ratiometric Depalmitoylation Probe 2 (RDP-2). (i) 20% TFA, DCM, N_2_, 1 h. (ii) EDC·HCl, cyanoacetic acid, DMF, 48 h. (iii) Lys(Boc)-OMe·HCl, EDC·HCl, HOBt, DIPEA, DMF, 3 h. (iv) Piperdine, EtOH : DCM 1 : 1, 72 h. (v) I_2_, MeOH, 30 min. (vi) TCEP, MeOH. (vii) Octanoic anhydride, Et_3_N, DMF. (viii) 15% TFA, DCM.

A multitude of fluorescent probes for biorelevant thiols have been developed,^23^–^26^ many of which rely on Michael-acceptor motifs reacting with the thiol of interest to produce a fluorescent response. Alkene and aldehyde modifications at the 3-position of aminocoumarins have been exploited for fluorescent sensors due to the often ratiometric fluorescent response of chemical alterations at this site on the fluorophore.^23^–^29^ We sought to leverage this design strategy by developing a Michael acceptor motif that does not react intermolecularly with glutathione, but does react intramolecularly with a pendant acylated cysteine APT substrate after deprotection. Based on this concept, we designed the RDPs, which feature a previously validated^15^*S*-palmitoyl-cysteine analog (*S*-octanoyl cysteine) residue, which increases water solubility, coupled to a weakly active Michael acceptor moiety^27^–^29^ at the 3-position on a julolidine-based amino coumarin fluorophore *via* a cyanoacetamide possessing an α,β-unsaturation. When the probe is processed by APTs, the now free cysteine thiol will undergo a Michael addition with the unsaturated portion, resulting in the breaking of the conjugated π-system thereby causing a change in the spectral properties of the probe (Scheme 1). Methylation at the 4-position should eliminate intermolecular nucleophilic attack at positions other than designed.^30^ Synthesis of RDP-1, the simplest probe with a methyl-amide C-terminal modification, proceeded smoothly over 3 steps (Fig. S1†). We found that methylating the amide on the Michael acceptor dramatically enhanced stability due to known decarboxylation mechanisms of cyanoacids.^31^,^32^ Additionally, we synthesized RDP-2 over 4 steps (Scheme 2), which features a C-terminal lysine modification to mimic known natural APT substrates, such as H-RAS,^1^,^33^ that we have previously found to enhance APT1 engagement on the DPP scaffold.^15^

### *In vitro* response of RDPs to purified APTs

**Fig. 1:**
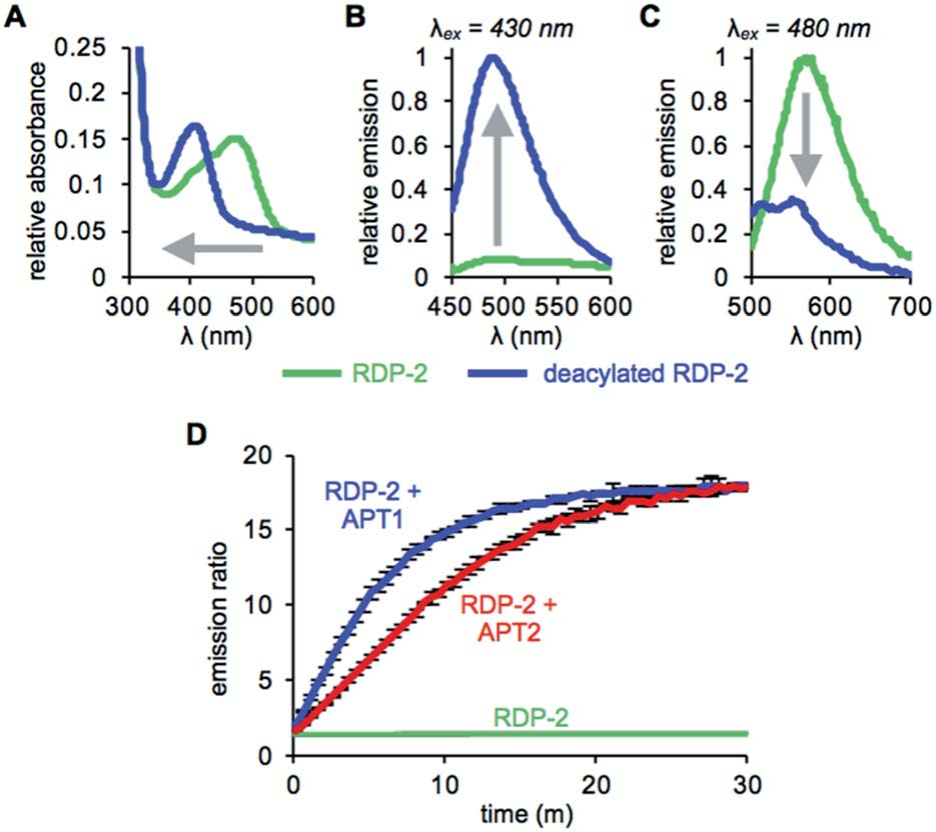
UV-Vis absorption (A) and fluorescence emission at *λ*_ex_ = 430 nm (B) and *λ*_ex_ = 480 nm (C) of 15 μM RDP-2 and deacylated RDP-2 in buffer (20 mM HEPES, 150 mM NaCl, 0.1% Triton X-100, pH 7.4). (D) Ratiometric response of 1 μM RDP-2 to 200 nM APT1 or 200 nM APT2. Error bars are ± std. dev.

With RDP-1 and RDP-2 in hand, we tested their photophysical properties *in vitro*. RDP-1 and RDP-2 have absorbance maxima at 470 and 473 nm, respectively (Fig. S2A† and 1A). Upon deacylation, the absorbance maxima dramatically shift to 406 and 407 nm, respectively, indicating that the Michael addition is occurring as designed. Illumination with either 430 nm or 480 nm light permits selective excitation of the two forms of the probe, with the fluorescence emission upon 430 nm excitation increasing upon deacylation (Fig. 1B and S2B†) and the fluorescence emission upon 480 nm excitation decreasing upon deacylation (Fig. 1C and S2C†). We used these ratiometric fluorescence responses to monitor enzymatic activity of RDP-1 and RDP-2 with purified human APT1 and APT2. Incubating 1 μM probe with 200 nM enzyme results in a rapid change in the ratiometric response, with a ratio change from 0.7 to 34 for RDP-1 (Fig. S2D†), and 1.9 to 16 for RDP-2 after just 20 min (Fig. 1D). Critically, neither RDP-1 or RDP-2 responds to catalytically inactive APTs (Fig. S3†) or 20 mM glutathione (Fig. S4†).

### RDPs can measure endogenous APTs in live cells

**Fig. 2:**
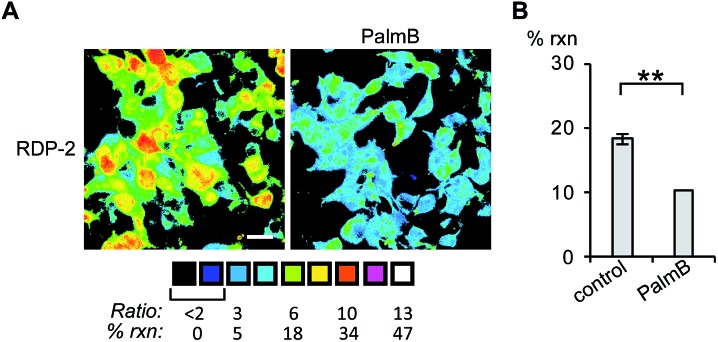
Ratiometric fluorescence imaging of RDP-2 in live HEK293T cells treated with PalmB. (A) Cells were treated with DMSO or 20 μM PalmB for 30 min, loaded with 1 μM RDP-2 for 10 min, and imaged. (B) Quantification of imaging in (A). **p < 0.005. Scale bar = 20 μm. Error bars are ± std. dev. (*n* = 3).

Having established that the RDPs respond to purified APTs *in vitro*, we next evaluated their ability to visualize endogenous *S*-depalmitoylase activity in living cells using fluorescence microscopy. Similar to the *in vitro* ratiometric fluorescence experiments, we monitored the redder emission (480/20 nm excitation, 575/40 nm emission) and bluer emission (430/24 nm excitation, 470/24 nm emission) simultaneously, and divided the intensities at both wavelengths to generate ratiometric images. We estimated the percent deprotected dye by assuming a linear relationship between the acylated (0%) and deacylated dye (100%). Treating data in this way has the benefit of allowing for the comparison of the probes in situations where there are differences in microscope settings day to day or between different cell or tissue types. Treatment of HEK293T cells with 1 μM RDP-1 or RDP-2 for 10 min results in *ca.* 28% and 18% depalmitolyation, respectively (Fig. 2, S5 and S6†). Importantly, loading HEK293T cells with 4, an analog of RDP-1 that cannot respond to depalmitoylation or any other cellular process, reports negligible (*ca.* 1%) deprotection, ruling out interference from other cellular nucleophiles (Fig. S7†). Pretreatment of the cells with the non-specific *S*-depalmitoylase inhibitor PalmB^20^ decreased the measured depalmitoylation activity almost in half for RDP-1 and RDP-2 (Fig. 2, S5 and S6†). Collectively, these results confirm that the RDPs are measuring endogenous *S*-deacylase activities.

**Fig. 3:**
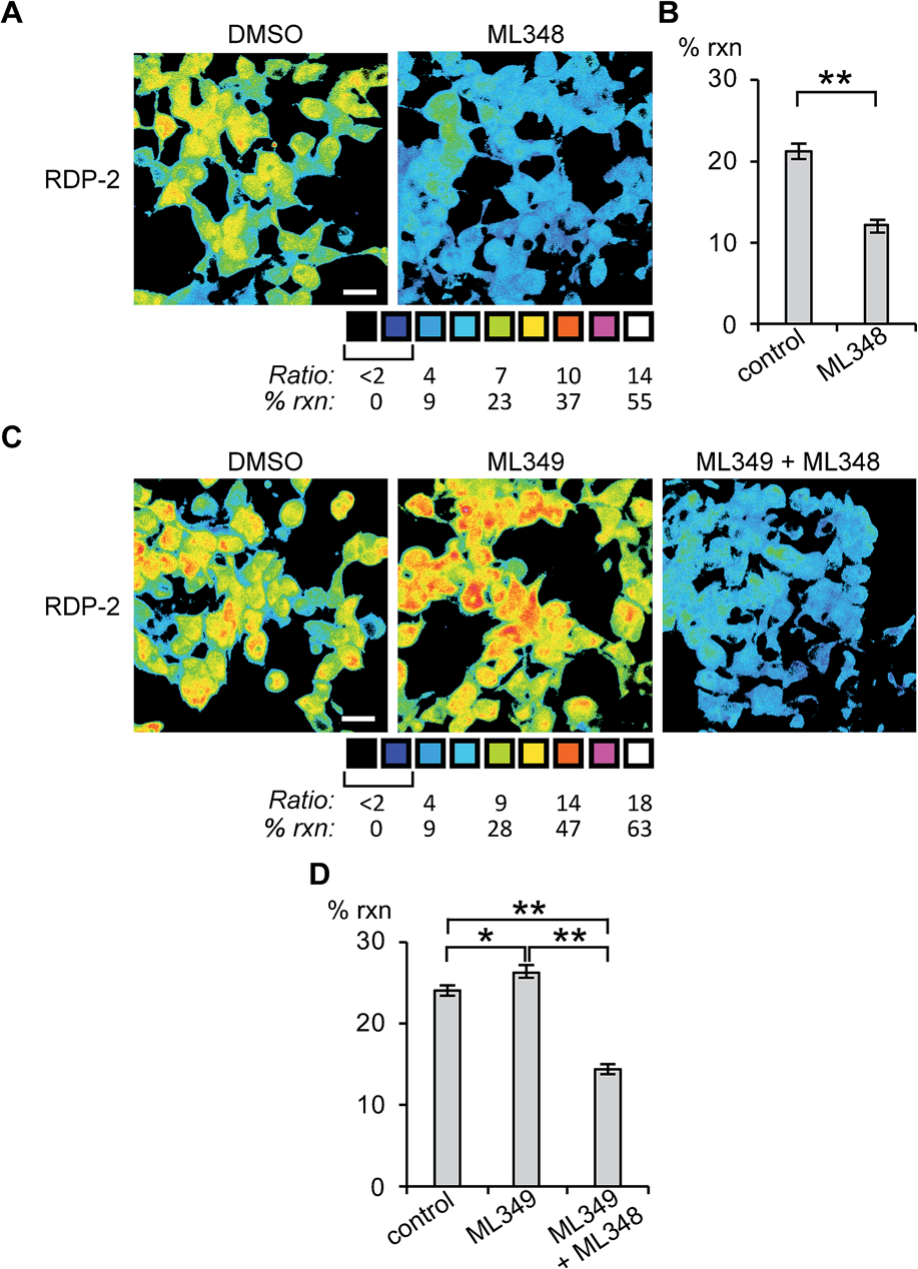
Ratiometric fluorescence imaging of RDP-2 in live HEK293T cells treated APT inhibitors. Cells were treated with (A) DMSO or 5 μM ML348 for 30 min and (C) DMSO, 5 μM ML349, or 5 μM ML349 and 5 μM ML348, loaded with 1 μM RDP-2 for 10 min, and imaged. Quantification of (B) ML348 and (D) ML349 with and without ML348 imaging. *p < 0.03, **p < 0.005. Scale bar = 20 μm. Error bars are ± std. dev. (*n* = 3).

Next, we sought to assess the effects of modulation of specific APT isoforms on the responses from the RDPs. Treating cells with the APT1 and APT2-selective inhibitors, ML348 and ML349,^33^,^34^ respectively, both displayed a slight increase in depalmitoylation activity from RDP-1 (Fig. S8 and S9†). In contrast, RDP-2 showed a *ca.* 30% decrease in depalmitoylation activity in ML348-treated HEK293T cells and an increase by *ca.* 12% upon ML349 treatment (Fig. 3, S10 and S11†). The increase in depalmitoylation of RDP-2 upon APT2 inhibition is reversed when both ML348 and ML349 are administered (Fig. 3B and D). We attribute the increase in depalmitoylation in response to specific APT inhibitors to possible compensatory mechanisms, including but not limited to changes in activity or localization of the APTs. For example, the APTs have been previously been found to autoregulate the palmitoylation states of one another.^35^,^36^ This compensation masks APT1/APT2 inhibition for the pan-depalmitoylase probe, RDP-1. However, RDP-2, which is more sensitive to changes in APT1 activity, shows an enhancement in signal upon APT2 inhibition. APT2 knockdown experiments with RDP-2 reveal that the compensation is transient, as knocking down APT1, which requires at least 24 h, decreases RDP-2 deacylation similar to the levels with ML348 (Fig. S12 and S13†).

### RDPs can measure endogenous APTs in human colon organoids

**Fig. 4:**
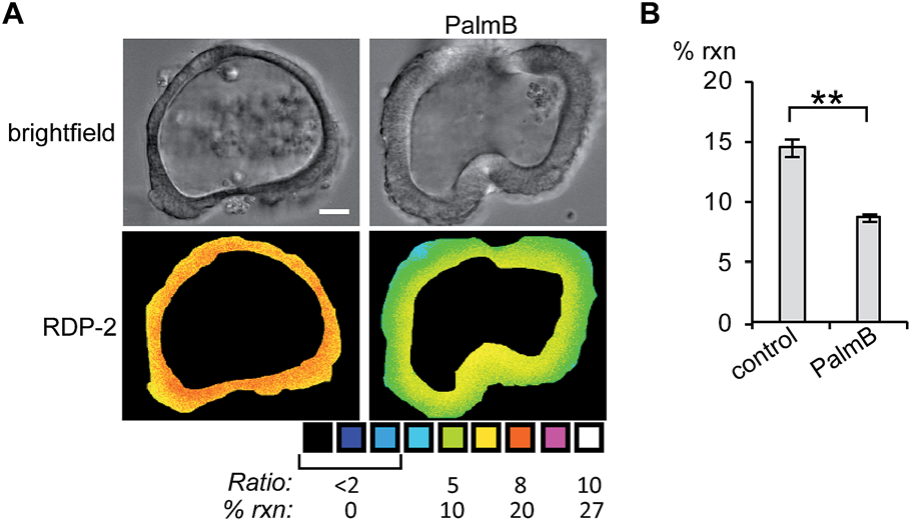
Ratiometric fluorescence imaging of RDP-2 in live human colon organoids cells treated with PalmB. (A) Organoids were treated with DMSO or 40 μM PalmB for 30 min, loaded with 5 μM RDP-2 for 10 min, and imaged. (B) Quantification of imaging in (A). **p < 0.005. Scale bar = 20 μm. Error bars are ± std. dev. (*n* = 3).

Next, we tested whether RDPs could also be deployed in heterogeneous primary tissue samples. We chose to test whether we could detect endogenous *S*-depalmitoylases in human colon organoids^37^ as an *ex vivo* model to study human metabolism. Imaging organoids is difficult due to the high level of heterogeneity inherent to the tissue, and therefore serves as a challenging testbed to assay our new probes. Colon organoids loaded with RDP-2 confirm that these human tissues have *S*-depalmitoylase activity (Fig. 4, S14 and S15†). Pretreating the organoids with PalmB inhibited the measured *S*-depalmitoylation by *ca.* 50%. Furthermore, we observed a *ca.* 20% decrease of in the *S*-depalmitoylase activity when the organoids were treated with ML348, suggesting APT1-like activity is present in the human colon (Fig. S16 and S17†). As observed by the high degree of structural heterogeneity between the organoids, these experiments are substantially enabled by the ratiometric nature of the RDPs (Fig. S14–S17†).

### RDPs can detect endogenous APT activity changes during lipid stress

**Fig. 5:**
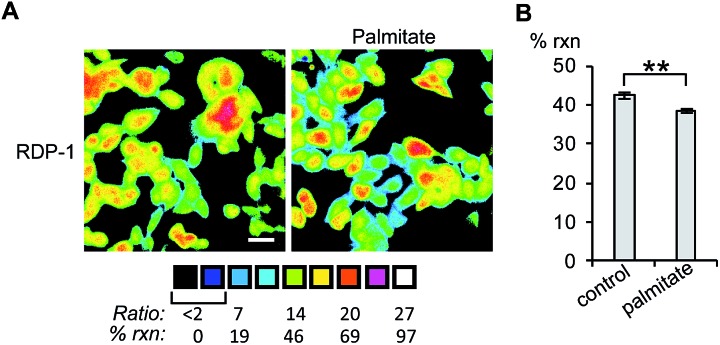
Ratiometric fluorescence imaging of RDP-2 in live HEPG2 cells treated with palmitate. (A) Cells were treated with 1% BSA as a control or 1 mM palmitate in 1% BSA for 6 h, loaded with 1 μM RDP-2 for 10 min, and imaged. (B) Quantification of imaging in (A). **p < 0.005. Scale bar = 20 μm. Error bars are ± std. dev. (*n* = 3).

Finally, we tested whether the RDPs could measure subtle changes to endogenous *S*-depalmitoylases during lipid stress conditions. Dysregulated fatty acid synthesis and stress has been observed in several diseases including cancer and non-alcoholic fatty liver disease.^22^,^38^,^39^ As the liver is a major center of production of fatty acids, HEPG2 cells were chosen as they should be particularly sensitive to changes in fatty acid pools.^22^ Additionally, treatment of HEPG2 with palmitate has been previously demonstrated to alter lipid metabolism and cause ER stress.^38^,^39^ PalmB treatment of HEPG2 cells displayed a *ca.* 60% decrease in deacylation of RDP-1 confirming we can monitor APT activity in HEPG2 cells (Fig. S18†). Pretreatment of HEPG2 cells with palmitate for 6 h resulted in a decrease in APT activity, as measured by RDP-1 (Fig. 5 and S19†). This experimentally validates a previously suggested link between cellular lipid status and *S*-depalmitoylation activity.

## Conclusions

In summary, we have presented RDPs, a new strategy for the development of fluorescent probes for cysteine PTM eraser proteins. RDP-1 and RDP-2 respond to the known cytosolic *S*-depalmitoylases *in vitro* and detect endogenous APTs in live cell culture models with a robust ratiometric response. Moreover, RDPs can monitor endogenous APTs in primary tissue samples, enabled by their ratiometric response. Finally, we deployed RDP-1 to demonstrate APT activity is linked to lipid metabolism. Current efforts are now underway to explore physiological signaling roles for APTs using RDPs in the human colon and other primary samples, as well as to expand the chemical approach to other cysteine PTMs.^40^,^41^

## Conflicts of interest

The authors have filed a provisional patent on the RDP scaffold.

## Supplementary Material

Supplementary information
