# Correction: Michael addition-based probes for ratiometric fluorescence imaging of protein *S*-depalmitoylases in live cells and tissues

**DOI:** 10.1039/c7sc90066j

**Published:** 2017-10-12

**Authors:** Michael W. Beck, Rahul S. Kathayat, Candace M. Cham, Eugene B. Chang, Bryan C. Dickinson

**Affiliations:** a Department of Chemistry , The University of Chicago , 5801 South Ellis Avenue , Chicago , Illinois 60637 , USA . Email: Dickinson@UChicago.edu; b Department of Medicine , The University of Chicago , 5801 South Ellis Avenue , Chicago , Illinois 60637 , USA

## Abstract

Correction for ‘Michael addition-based probes for ratiometric fluorescence imaging of protein *S*-depalmitoylases in live cells and tissues’ by Michael W. Beck *et al.*, *Chem. Sci.*, 2017, DOI: ; 10.1039/c7sc02805a



## 

The authors regret that there was an error in ref. 30 of the original article. The correct reference, including the two relevant journals, is presented herein as ref. 1.

The Royal Society of Chemistry apologises for these errors and any consequent inconvenience to authors and readers.
