# Supplementary material for: Michael addition-based probes for ratiometric fluorescence imaging of protein S-depalmitoylases in live cells and tissues
Source: Chem Sci. 2017 Sep 11;8(11):7588–92. doi: 10.1039/c7sc02805a (PMC5848818; doi:10.1039/c7sc02805a)
Supplement: Supplementary file 1 [file SC-008-C7SC02805A-s001.pdf]

Electronic Supplementary Material (ESI) for Chemical Science.
This journal is © The Royal Society of Chemistry 2017

†ELECTRONIC SUPPORTING INFORMATION:

Michael addition-based probes for ratiometric fluorescence imaging of protein *S*-depalmitoylases in live cells and tissues

Michael W. Beck,[†] Rahul S. Kathayat,[†] Candace M. Cham,[‡] Eugene B. Chang,[‡] Bryan C. Dickinson[*,†]

[†]Department of Chemistry, [‡]Department of Medicine, The University of Chicago, 5801 South Ellis Avenue, Chicago, Illinois, 60637, United States

*Corresponding author. Email address: Dickinson@uchicago.edu (Bryan C. Dickinson)

Synthetic materials and methods. Silica gel P60 (SiliCycle, 40-63 μm, 230-400 mesh) was used for column chromatography. Analytical thin layer chromatography was performed using SiliCycle 60 F254 silica gel (precoated sheets, 0.25 mm thick). All chemicals for synthesis were purchased from Sigma-Aldrich (St. Louis, MO) or Fisher Scientific (Pittsburgh, PA) and used as received. ML348 and ML349 were purchased from Tocris (Bristol, UK). ^{1}H-NMR and ^{13}C-NMR spectra were collected in NMR solvents $CDCl_3$/$(CD_3)_2CO$ (Sigma-Aldrich,) at 25 ºC using a 500 MHz Bruker Avance II+ spectrometer with 5 mm QNP probe at the Department of Chemistry NMR Facility at the University of Chicago. ^{1}H-NMR chemical shifts are reported in parts per million (ppm) relative to the peak of residual proton signals from ($CDCl_3$ 7.26 ppm or $(CD_3)_2CO$ 2.05 ppm). Multiplicities are given as: s (singlet), d (doublet), t (triplet), q (quartet), dd (doublet of doublets), m (multiplet). ^{13}C-NMR chemical shifts are reported in parts per million (ppm) relative to the peak of residual proton signals from ($CDCl_3$ 77.16 ppm or $(CD_3)_2CO$ 29.84 ppm amd 206.26 ppm). Analysis of NMR and plots were obtained using TopSpin 3.5 pl 7 (Bruker, Billerica, MA). High resolution mass was obtained from Agilent 6224 TOF High Resolution Accurate Mass Spectrometer (HRA-MS) using combination of APCI and ESI at the Department of Chemistry Mass Spectrometry Facility at the University of Chicago. Low resolution mass spectral analyses and liquid chromatography analysis were carried out on an Advion Expression-L mass spectrometer (Ithaca, NY) coupled with an Agilent 1220 Infinity LC System (Santa Clara, CA). All experiments utilizing a plate reader were carried out on a BioTek (Winooski, VT) monochromator-based Synergy Neo2 Multi-Mode Reader at 37 ºC. IUPAC names for compounds were generated using MarvinSketch (Version 17.8.0, ChemAxon, Budapest, Hungary). *tert*-butyl (*R*)-methyl(1-(methylamino)-1-oxo-3-(tritylthio)propan-2-yl)carbamate (1)[1] and *N*-(*tert*-butoxycarbonyl)-*N*-methyl-*S*-trityl-L-cysteine (5)[1, 2] were obtained as previously reported. Protocols for obtaining human tissue samples were approved by the IRB of the University of Chicago (approval 15573A), and all patients provided written informed consent.

Synthesis of 2 ((2R)-2-(2-cyano-N-methylacetamido)-N-methyl-3-[(triphenylmethyl)-sulfanyl]propanamide). In a vial sealed with a rubber septa and equipped with a stir bar, **1** (0.2542 g, 0.5 mmol) was dissolved in 5 mL CH_2Cl_2 and placed under a nitrogen atmosphere. Trifluoroacetic acid was slowly added until the concentration was *ca*. 20% v/v and allowed to stir for 1 h. After complete boc-deprotection as determined by LC-MS, the mixture was concentrated and then redissolved in CH_2Cl_2 (50 mL) and washed with sat. bicarbonate (50 mL). The organic layer was dried over sodium sulfate, filtered, and concentrated in a round bottom flask. A stirbar, cyanoacetic acid (0.1322 g, 1.5 mmol), EDC•HCl (0.9935 g, 5.2 mmol), and hoBt (0.2381 g, 1.5 mmol) were added to the flask, sealed with a rubber septa, and placed under a flow of nitrogen. The flask was then cooled to 0 ºC in an ice bath before adding DMF *ca*. 15

mL and DIPEA (0.27 mL, 1.5 mmol). The reaction mixture was allowed to slowly warm to room temperature and react overnight. The following day, the reaction was diluted with brine (50 mL) and extracted into EtOAc (3x75 mL). The organic layers were combined, dried over sodium sulfate, filtered, and concentrated. Purification by flash column chromatography (gradient of 0% to 100% EtOAc in Hexanes) yielded **2** with quantitative yield. R_f (70% EtOAc in Hexanes) = 0.39. ^{1}H-NMR (500 MHz; $CDCl_3$) δ: 7.42 (d, *J* = 10, 6H), 7.30 (t, *J* = 10, 6H), 7.23 (t, *J* = 10, 3H), 5.71 (s, 1H), 4.50 (m, 1H), 3.47 (s, 2H), 2.77 (m 4H), 2.68 (m, 3H), 2.65 (m, 1H). ^{13}C-NMR (126 MHz; $CDCl_3$) δ: 168.8, 144.3, 129.6, 129.5, 128.4, 128.2, 127.3, 127.0, 67.2, 56.4, 29.9, 26.3, 25.7. HRA-MS(+) Calculated for $C_{27}H_{27}N_3O_2S$ [M^+] 457.1824; found 457.1839.

Synthesis of 3 (6-methyl-4-oxo-3-oxa-13-azatetracyclo[7.7.1.0^{2,7}.0^{13,17}]heptadeca-1,5,7,9(17)-tetraene-5-carbaldehyde). A previously-reported procedure was adapted for the synthesis of **3.**[3] To a flame-dried vial equipped with a stirbar, 2,3,6,7-Tetrahydro-9-methyl-1*H*,5*H*-quinolizino[9,1-gh]coumarin[4] (0.6064 g, 2.4 mmol) was dissolved in a minimal amount of dry DMF (*ca*. 4 mL) under a nitrogen atmosphere. $POCl_3$ (6.8 mmol, 0.63 mL) was added dropwise over 5 min. The resulting solution was allowed to stir for 1 h until complete by LC-MS. The reaction was then quenched by dilution with 5 mL CH_2Cl_2, followed by sat. bicarbonate solution until gas evolution ceased. The resulting mixture was diluted with 50 mL brine extracted into CH_2Cl_2 (3x 100 mL). The organic layer was dried over sodium sulfate, filtered, and concentrated. Purification by flash column chromatography (gradient of 0% to 5% MeOH in CH_2Cl_2) yielded **3** (0.544 g, 80.8%) as an orange solid in 80.8% yield. R_f (5% MeOH in DCM) = 0.50. ^{1}H-NMR (500 MHz; $CDCl_3$) δ: 10.37 (s, 1H), 7.24 (s, 1H), 3.34 (m, 4H), 2.88 (t, *J* = 10 Hz, 2H), 2.78 (m, 5H), 1.98 (m, 4H). ^{13}C-NMR (126 MHz; $CDCl_3$) δ: 191.6, 163.3, 159.3, 152.3, 148.6, 124.6, 119.4, 111.5, 109.1, 106.0, 50.3, 49.9, 27.8, 21.3, 20.3, 20.2, 14.9. HRA-MS(+) Calculated for $C_{17}H_{17}NO_3$ [M^+] 283.1208; found 283.1243.

Synthesis of 4 ((2E)-2-cyano-N-methyl-3-{6-methyl-4-oxo-3-oxa-13-azatetracyclo [7.7.1.0^{2,7}.0^{13,17}]heptadeca-1,5,7,9(17)-tetraen-5-yl}-N-[(1R)-1-(methylcarbamoyl)-2-[(triphenylmethyl)sulfanyl]ethyl]prop-2-enamide). In a flame-dried vial equipped with a stir bar, **3** (0.1140 g, 0.4 mmol) and **2** (0.1843 g, 0.4 mmol) were dissolved in 10 mL 9:1 dry CH_2Cl_2:dry EtOH followed by two drops of piperdine. The vial was then capped and allowed to stir at room temperature for 72 h. When complete as determined by LC-MS, the reaction mixture was concentrated and purified by flash column chromatography (gradient of 0% to 10% MeOH in CH_2Cl_2) to yield a mixture of both the Z and E isomers of **4** (0.127 g, 99.6%) as a red solid, which were used without farther purification. R_f (10% MeOH in CH_2Cl_2) = 0.67. ^{1}H-NMR (500 MHz, $[CD_3]_2CO$): 7.63 (s, 1H), 7.46 (d, *J* = 10 Hz, 6H), 7.37 (s, 1H), 7.34 (t, *J* = 10 Hz, 6H),

7.25 (m, 3 H), 4.73 (m, 1H), 3.38 (m, 6H), 2.94 (m, 4H), 2.80 (m, 4H), 2.69 (d, *J* = 5 Hz, 3H), 2.56 (s, 3H), 1.96 (m, 4H). ^{13}C-NMR (126 MHz; $CDCl_3$) δ: 191.2, 168.9, 164.6, 159.8, 154.4, 150.6, 147.7, 144.6, 143.6, 129.5, 127.8, 126.5, 123.3, 119.4, 116.9, 110.3, 109.8, 108.8, 108.5, 105.6, 105.4, 66.6, 50.0, 49.5, 29.9, 27.6, 26.2, 21.0 (Extra peaks present likely due to mixture of Z and E isomers). HRA-MS(+) Calculated for $C_{44}H_{42}N_4O_4S$ [M^+] 722.2927; found 722.2934.

Synthesis of RDP-1 ((2E)-2-cyano-N-methyl-3-{6-methyl-4-oxo-3-oxa-13-azatetracyclo [7.7.1.0^{2,7}.0^{13,17}]heptadeca-1,5,7,9(17)-tetraen-5-yl}-N-[(1R)-1-(methylcarbamoyl)-2-(octanoyl sulfanyl)ethyl]prop-2-enamide). In a vial, **4** (0.1272 g, 0.1 mmol) was dissolved in 3 mL of MeOH, followed by addition of iodine (0.0370 g, 0.2 mmol). The solution was stirred for 30 min before quenching with a 10 mL solution containing 0.2 M sodium citrate and 0.2 M sodium ascorbate in H_2O at pH 4.0. The resulting mixture was extracted into CH_2Cl_2 (3x 30mL). The organic layers were combined, dried over sodium sulfate, filtered, concentrated, semi-purified by flash column chromatography (gradient of 0% to 10% MeOH in CH_2Cl_2), and used without further purification. The resultant red residue was dissolved in 3 mL dry DMF in a vial under a nitrogen atmosphere, to which DTT (.0472 g, 0.4 mmol) and then 0.05 mL of DIPEA (0.4 mmol) were added, after which the red solution turned yellow. After 5 min, product formation was confirmed by LC-MS and the solution was diluted with 1 M HCl (10 mL) and extracted into EtOAc (3x 20 mL). The organic layers were combined, dried over sodium sulfate, filtered, and concentrated. The resulting orange-yellow oil was dissolved in 3 mL DMF and octanoic anhydride (0.72 mL, 2.4 mmol) was added immediately followed by the dropwise addition of DIPEA (0.42 mL, 2.4 mmol). After 30 min, the red solution was diluted with 30 mL brine and extracted into CH_2Cl_2 (3X 30 mL). The organic layers were combined, dried over sodium sulfate, filtered, concentrated, and purified by flash column chromatography (first column gradient of 0% to 100% CH_2Cl_2 in hex to 10% MeOH in CH_2Cl_2, second column 0% to 100% EtOAc in hex) to yield **RDP-1** as a red solid (0.0332 g, 44.7%). R_f (5% MeOH in CH_2Cl_2) = 0.47. ^{1}H-NMR (500 MHz, $[CD_3]_2CO$): 7.70 (s, 1H), 7.29 (s, 1H), 5.11 (s, 1H), 4.97(s, 1H), 3.36 (m, 6H), 3.30 (s, 1H), 3.07 (s, 1H), 2.78 (m, 10H), 2.59 (t, *J* = 5 Hz), 2.54 (m, 2H), 2.47 (s, 3H), 1.97 (m, 7H), 1.62 (m, 4H) HRA-MS(+) Calculated for $C_{33}H_{42}N_4O_5S$ [M^+] 606.2876; found 606.2874. Purity ascertained by LC-MS.

Synthesis of 6 (methyl (2S)-6-{[(tert-butoxy)carbonyl]amino}-2-[(2R)-2-(2-cyano-N-methylacetamido)-3-[(triphenylmethyl)sulfanyl]propanamido]hexanoate). In a vial sealed with a rubber septa and equipped with a stir bar, 5[1, 2] (2.0000 g, 4.2 mmol) was dissolved in 10 mL CH_2Cl_2 and placed under a nitrogen atmosphere. Trifluoroacetic acid was slowly added until the concentration was *ca*. 20% v/v and allowed to stir for 1 h. After complete boc-deprotection as

determined by LC-MS, the mixture was concentrated and then dissolved in CH_2Cl_2 (50 mL) and dried by rotary evaporation. Dissolving and drying was then repeated two additional times. After the last time, the vial was dried *in vacuo* and in a separate flame dried flask sealed with a septa and under nitrogen atmosphere cyanoacetic acid (1.8026 g, 21.2 mmol) and EDC•HCl (1.0156 g, 5.3 mmol) were dissolved in dry DMF (15 mL). After 1 h, 1.0000 g (2.6 mmol) of the boc-deprotected cysteine was added to the mixture of EDC and cyanoacetic acid followed by the addition of DIPEA (0.92 mL, 5.3 mmol). The resulting solution was then stirred at rt for 48 h before being diluted with brine (100 mL) and extracted into CH_2Cl_2 (3x 100 mL). The organic layers were combined, dried over sodium sulfate, filtered, concentrated, and purified by flash column chromatography (0% to 10% MeOH in 1:1 EtOAc:CH_2Cl_2) and used without further purification. R_f (10% MeOH in CH_2Cl_2) = 0.38. Under a nitrogen atmosphere in a flame dried vial sealed with a septa equipped with a stir bar, 1.0000 g of the resulting oil, (2.2 mmol), NH_2-Lys(Boc)-OMe•HCl (1.0014 g, 3.3 mmol), EDC•HCl (4.3122 g, 22.5 mmol), and hoBt (1.0334 g, 6.7 mmol) were cooled down to 0 °C in an ice bath before the addition of DMF (40 mL) and DIPEA (1.2 mL, 6.7 mmol). After 5 minutes of stirring at 0 °C the solution was allowed to warm up to rt. After 3 h, when complete as determined by LC-MS, the solution was diluted with brine (100 mL) and extracted into EtOAc (3x 100 mL). The organic layers were combined, dried over sodium sulfate, filtered, concentrated, and purified by flash column chromatography (0% to 10% MeOH in CH_2Cl_2, 2x) to yield **7** (0.3770 g, 24.4%). R_f (50% EtOAc in hexanes) = 0.33. ^{1}H-NMR (500 MHz, $CDCl_3$): 7.42 (m, 6H), 7.30 (m, 6H), 7.22 (m, 3H), 6.87 (s, 1H), 4.56 (m, 5H), 4.39 (m, 1H), 3.76 (s, 5H), 3.69 (s, 2H), 3.44 (s, 3H), 3.10 (m, 3H), 2.78 (s, 2H), 1.41 (s, 9H). ^{13}C-NMR (126 MHz; $CDCl_3$) δ: 172.2, 172.1, 161.7, 156.3, 144.2, 129.5, 129.4, 128.3, 128.1, 127.2, 126.9, 114.6, 79.2, 67.1, 56.4, 52.8, 52.6, 52.4, 52.1, 29.5, 28.4, 25.8 HRA-MS(+) Calculated for $C_{38}H_{46}N_4O_6S$ [M^+] 686.3138; found 686.3142

Synthesis of 7 (methyl (2S)-6-{[(tert-butoxy)carbonyl]amino}-2-[(2R)-2-[(2E)-2-cyano-N-methyl-3-{6-methyl-4-oxo-3-oxa-13-azatetracyclo[7.7.1.0^{2,7}.0^{13,17}]heptadeca-1,5,7,9(17)-tetraen-5-yl}prop-2-enamido]-3-[(triphenylmethyl)sulfanyl]propanamido]hexanoate). In a flame-dried vial equipped with a stir bar, **6** (0.2426 g, 0.4 mmol) and **2** (0.1000 g, 0.4 mmol) were dissolved in 10 mL 9:1 dry CH_2Cl_2:dry EtOH followed by two drops of piperdine. The vial was then capped and allowed to stir at room temperature. After 48 h, an addition 0.1000 g of **2** (0.4 mmol) was added and it was allowed to stir for 48 h longer. When complete as determined by LC-MS, the reaction mixture was concentrated and purified by flash column chromatography (First column gradient of 0% to 30% EtOAc in CH_2Cl_2, second column gradient of 0% to 100% EtOAc in Hexanes) to yield a mixture of both the Z and E isomers of **8** (0.0710 g, 21.1%) as a red solid, which were used without farther purification. R_f (50% EtOAc in hexanes) = 0.28. ^{1}H-

NMR (500 MHz, $[CD_3]_2CO$):7.63 (s, 1H), 7.46 (m, 6H), 7,38 (s, 1H), 7.33 (m, 6H), 7.24 (m, 3H), 4.96 (m, 1H), 4.78 (s, 2H), 4.34 (m, 2H), 4.24 (m, 1H), 3.68 (m, 1H), 3.62 (s, 1H), 3.38 (m, 2H), 3.30 (m, 1H), 2.99 (m, 4H), 2.81 (m, 3H), 2.77 (m, 1H), 2.57 (m, 1H), 2.52 (m, 1H), 2.45 (m, 1H), 1.96 (m, 6H), 1.50 (m, 3H), 1.40 (m, 3H), 1.34 (m, 9H), 1.29 (m, 1H) ^{13}C-NMR (126 MHz; $[CD_3]_2CO$) δ: 173.0947, 169.116, 165.3539, 160.8237, 155.8995, 151.5859, 151.4283, 148.6708, 148.1291, 145.6671, 144.6527, 130.3529, 128.8953, 128.787, 127.7135, 127.5264, 124.5226, 120.5734, 117.7371, 111.9364, 111.2766, 110.8531, 109.238, 109.1001, 106.1259, 78.2747, 67.2249, 53.9198, 52.1372, 50.6403, 50.0986, 40.7131, 31.2981, 30.9337, 28.6489, 28.2352, 24.1974, 22.0012, 21.8831, 20.849, 20.6717, 15.649 (extra peaks present likely due to mixture of Z and E isomers). HRA-MS(+) Calculated for $C_{55}H_{61}N_5O_8S$ [M^+] 951.4241; found 951.4222

Synthesis of RDP-2 ((5S)-5-[(2R)-2-[(2E)-2-cyano-N-methyl-3-{6-methyl-4-oxo-3-oxa-13-azatetracyclo[7.7.1.0^{2,7}.0^{13,17}]heptadeca-1,5,7,9(17)-tetraen-5-yl}prop-2-enamido]-3-(octanoylsulfanyl)propanamido]-6-methoxy-6-oxohexan-1-aminium). Iodine (0.0379 g, 0.02 mmol) was added in one portion to a mixture of **8** (0.0710 g, 0.01 mmol) and 1 mL MeOH in a vial equipped with a stirbar. After 30 mins, the solution was quenched with a solution of 0.2 M sodium citrate and 0.2 M sodium ascorbate in water at pH 4.0 (5 mL) and extracted into CH_2Cl_2 (3x 15 mL). The organic layers were combined, dried over sodium sulfate, filtered, and concentrated before redissolving in MeOH (1 mL) and a solution of (tris(2-carboxyethyl)phosphine (0.4278 g, 1.5 mmol) in MeOH 1 mL and 0.5 mL water was added. After 2 h, the reaction mixture was concentrated *in vacuo* and dissolved in 1 mL DMF. Octanoic anhydride (0.22 mL, 0.75 mmol) was then added followed by trimethylamine dropwise over 10 mins (0.10 mL, 0.75 mmol). After 30 mins, the solution was diluted with brine (5 mL) and the pH was adjusted to 8 with 1 M NaOH before extracting into CH_2Cl_2 (3x 10 mL). The organic layers were combined, dried over sodium sulfate, filtered, concentrated, and purified by flash column chromatography (first column gradient of 0% to 20% MeOH in CH_2Cl_2, second column gradient of 0% to 100% EtOAc in hexanes, third column gradient of 0% to 5% MeOH in CH_2Cl_2) R_f (5% MeOH in CH_2Cl_2) = 0.38. HRA-MS(+) Calculated for $C_{44}H_{61}N_5O_9S$ [M+] 835.4190; found 835.4197. The resulting oil was then dissolved in 0.5 mL DCM containing 15% v/v TFA and stirred for 30 min at room temperature. The solution was then concentrated and purified by flash column chromatography (first column gradient of 0% to 20% MeOH in CH_2Cl_2, second column gradient of 0% to 10% MeOH in CH_2Cl_2) yielding 0.0042 g (6.3%) of red oil. R_f (5% MeOH in CH_2Cl_2) = 0.50. HRA-MS(+) Calculated for $C_{39}H_{54}N_5O_7S$ [M^+] 736.3744; found 736.3807. Purity ascertained by LC-MS.

Purification of APT1 and APT2. Recombinant human APT1, APT2, APT1(S119A), and APT2(S122A) were purified using previously reported methods.[1] Purified proteins were stored in individual aliquots in protein storage buffer (50 mM Tris-HCl, pH 7.5, 300 mM NaCl, 1 mM DTT, 10% Glycerol) at 80 ºC. A fresh aliquot of protein was thawed for each experiment as we observed a time-dependent decline in activity of APT1 and APT2 once thawed.

***In vitro* kinetic assays of RDP-1 and RDP-2.** 100 μL of 400 nM of APT1 or APT2, or buffer without added enzymes, were added to buffer (20 mM HEPES, 150 mM NaCl, 0.1% Triton X-100, pH 7.4, degassed by bubbling N_2 through the solution for 1 h) and prewarmed to 37 ºC in a heating block for 5 min. The buffer solutions were then added using a multichannel pipet to a black-walled clear-bottom 96-well plate (Nunc) containing 100 μL of 2 μM **RDP-1** or **RDP-2** in buffer (20 mM HEPES, 150 mM NaCl, 0.1% Triton X-100, pH 7.4, 0.15% DMSO) that had been pre-warmed in the plate reader at 37 ºC for 5 min, resulting in a final concentration of 200 nM APT1 or APT2, 1 μM **RDP-1** or **RDP-2**, and 0.1% DMSO. Fluorescent intensities λ_{ex1}= 430/24 nm, λ_{em1} = 470/24 nm; λ_{ex2} = 480/20, λ_{em2} = 575/40; gain = 75; No. of flashes = 8) were obtained as a ratio of em1/em2 in kinetic mode in 30 s intervals for 30 min. For the catalytically inactive enzyme experiments, 10-times more APT1(S119A) and APT2(S122A) enzyme were used.

Stability of RDP-1 and RDP-2 with biological reductant. RDP-1 or **RDP-2** were added to buffer (20 mM HEPES, 150 mM NaCl, 0.1% Triton X-100, pH 7.4, degassed by bubbling N_2 through the solution for 1 h) prewarmed to 37 ºC in a heating block for 5 min containing no reducing agent or GSH using a multichannel pipet to at least three wells of a black-walled clear-bottom 96-well plate (Nunc; Final concentration: 1 μM **RDP-1/RDP-2**, 20 mM GSH, 20 mM HEPES, 150 mM NaCl, 0.1% Triton X-100, pH 7.4, 4.1% DMSO). Fluorescent intensities (λ_{ex1}= 430/25 nm, λ_{em1} = 470/24 nm; λ_{ex2} = 480/20, λ_{em2} = 575/40; gain = 75; No. of flashes = 10) were obtained as a ratio of em_1/em_2 in kinetic mode in 30 s intervals for 30 min.

UV-Vis and fluorescence spectra. A 30 μM solution of **RDP-1** or **RDP-2** in buffer (20 mM HEPES, 150 mM NaCl, 0.1% Triton X-100, pH 7.4, 0.15% DMSO) was diluted in half with buffer or buffer containing DTT (20 mM HEPES, 150 mM NaCl, 0.1% Triton X-100, pH 7.4, 40 mM DTT) to chemically deacylate the probe. After 1 h incubation at room temperature, 150 μL of the solutions were placed in a black-walled glass-bottom 96-well plate (Celvis, Mountain View, CA) and UV-Vis measurements (No. of flashes = 8, step size = 1 nm), fluorescent emission (λ_{Ex1} = 430/24 nm, λ_{Em1} = 450/5 nm to 800 nm, step size = 1 nm, gain = 57, No. of flashes = 10; λ_{Ex2} = 480/20 nm, λ_{Em2} = 500/5 nm to 800 nm, step size = 1 nm, gain = 95, No. of

flashes = 10), and fluorescent exicitation spectra (λ_{Ex1} = 300/24 nm to 450 nm, λ_{Em1} =470/5 nm, step size = 1 nm, gain = 57, No. of flashes = 10; λ_{Ex2} = 400/20 nm to 550 nm, λ_{Em2} = 575/5 nm, step size = 1 nm, gain = 95, No. of flashes = 10) were obtained on a plate reader.

Cell culture and maintenance. HEK293T (ATCC) and HEPG2 (gift from Prof. Chuan He) cells were maintained in DMEM/High Glucose (10% FBS, 1% P/S, L-Glutamine, Sodium pyruvate, Gibco) with 10% FBS (Gibco/Life Technolgies, Qualified US origin) at 37 °C and 5% CO_2. The cells were then used for less than 25 passages for all experiments. Multiple biological replicates were performed with cells from different passages and freshly thawed aliquots.

Colon organoids culture and maintenance. Generation of human colon organoids was performed as described by Sato *et. al.*[5] with some modifications. Briefly, pinch biopsies from colons of healthy human subjects were collected and washed three times with ice-cold PBS before an incubation at 4 ºC in 8 mM EDTA/PBS for 30 min, with gentle agitation. Individual crypts were released from the tissue by repeated tituration through a P1000 tip. Crypts and tissue were passed through a 100 um cell strainer (Falcon 352360). Crypts were then transferred to a microfuge tube and centrifuged at 300g for 5 min. Supernatant was discarded and pellets were resuspended in human organoid growth media [Advanced DMEM/F12, Glutamax, 1X HEPES buffer, Pen/Strep, N2 supplement, B-27 Supplement Minus Vitamin A (Thermo Fisher), 1.25 mM N-acetyl-L-(+)-cysteine, 10 mM Nicotinamide, 50% L-WRN conditioned media (L-WRN cells were a generous gift from T. C. He, Department of Surgery, The University of Chicago), murine epidermal growth factor (50 ng/mL; Peprotech, Rocky Hill, NJ), recombinant human R-spondin1 (500 ng/mL; Peprotech), Jagged-1 (1 μM; Anaspec, Fremont, CA), 10 μM Y-27632, 30 μM SB202190, 500 nM A-8301, 2.5 μM Chir99021, 500 nM LY2157299 (all chemical compounds from Cayman Chemical, Ann Arbor, MI), 10 nM Leu15-Gastrin I and 10 μg/mL ciprofloxacin (Sigma Aldrich)]. Organoids were mixed with Matrigel (Corning 356253) in a 1:2 ratio, plated onto pre-warmed 6-well tissue culture plates (Corning 3506), and placed in a 37 ºC/5% CO_2 incubator. After an hour, 2 mL organoid growth media was added. Organoids were maintained in culture for several weeks with media changes every 2-3 days.

Fluorescence imaging of RDP-1 and RDP-2 with HEK293T Cells. HEK293T were plated with 500 mL of DMEM (Gibco, High Glucose, Glutamax, 10% FBS) a four well chambered imaging dish (D35C4-20-1.5-N, Cellvis) which were precoated with 4 μg Poly-D-lysine (30-70 KDa, Alfa Aesar) for 2 h at room temperature or overnight at 4 ºC and grown to *ca.* 80% confluence prior to imaging. 30 min before the addition of the RDP, the cell culture media was removed and cells were washed with 500 μL of serum-free DMEM (Gibco, High Glucose,

Glutamax, no FBS). The media was then replaced with 500 μL of serum-free DMEM containing 100 nM Mitotracker Deep Red (ThermoFisher; to assist in focusing and processing) and either 0.1% DMSO carrier as a control or Palm B, ML348, or ML349 in DMSO and incubated for 30 min at 37 °C and 5% CO_2. The media was removed, the cells were washed with 500 μL of Live Cell Imaging Solution (ThermoFisher), and 500 μL of 1 μM **RDP-1** or **RDP-2** along with the appropriate inhibitor or DMSO as control in Live Cell Imaging Solution was added to each well. After incubation for 10 min at 37 °C and 5% CO_2, the cells were washed with 500 μL Live Cell Imaging Solution and 500 μL of Live Cell Imaging Solution containing DMSO or the appropriate inhibitor was added to the cells immediately before images were obtained on an inverted epi-fluorescence microscope (Olympus IX83) attached with EMCCD camera (Photometrics Evolve Delta) with 40x oil objective (N/A 1.3) for **RDP-1** (excitation filter 1 430/24 nm, dichroic chroma 89007, emission filter 1 470/24 nm, exposure time 100 ms, EM gain 80, excitation filter 2 480/20 nm, dichroic chroma 49014, emission filter 2 575/40 nm, exposure time 70 ms, EM gain 50) or for **RDP-2** (excitation filter 1 430/24 nm, dichroic chroma 89007, emission filter 1 470/24 nm, exposure time 70 ms, EM gain 50, excitation filter 2 480/20, dichroic chroma 49014, emission filter 2 575/40 nm, exposure time 50 ms, EM gain 30), brightfield (exposure time 50 ms, EM gain 30), and MitoTracker Deep Red: (excitation filter 640/30 nm, emission filter 705/72 nm, dichroic chroma 89016, exposure time 15 ms, EM gain 15). Analyses were performed in ImageJ (Wayne Rasband, NIH) as described below. Each experiment was repeated at least three separate times with identical results.

Fluorescence imaging of RDP-1 and 4 with HEK293T Cells. HEK293T cells were plated with 500 mL of DMEM (Gibco, High Glucose, Glutamax, 10% FBS) a four well chambered imaging dish (D35C4-20-1.5-N, Cellvis) which were precoated with 4 μg Poly-D-lysine (30-70 KDa, Alfa Aesar) for 2 h at room temperature or overnight at 4 ºC and grown to *ca*. 80% confluence prior to imaging. 30 min before the addition of the RDP, the cell culture media was removed and cells were washed with 500 μL of serum-free DMEM (Gibco, High Glucose, Glutamax, no FBS). The media was then replaced with 500 μL of serum-free DMEM containing 100 nM Mitotracker Deep Red (ThermoFisher; to assist in focusing and processing) and incubated for 30 min at 37 °C and 5% CO_2. The media was removed, the cells were washed with 500 μL of Live Cell Imaging Solution (ThermoFisher), and 500 μL of 1 μM **RDP-1** or 1 μM of 4. After incubation for 10 min at 37 °C and 5% CO_2, the cells were washed with 500 μL Live Cell Imaging Solution and 500 μL of Live Cell Imaging Solution was added to the cells immediately before images were obtained on an inverted epi-fluorescence microscope (Olympus IX83) attached with EMCCD camera (Photometrics Evolve Delta) with 40x oil objective (N/A 1.3; excitation filter 1 430/24 nm, dichroic chroma 89007, emission filter 1 470/24 nm, exposure time

100 ms, EM gain 50, excitation filter 2 480/20 nm, dichroic chroma 49014, emission filter 2 575/40 nm, exposure time 70 ms, EM gain 50), brightfield (exposure time 30 ms, EM gain 30), and MitoTracker Deep Red: (excitation filter 640/30 nm, emission filter 705/72 nm, dichroic chroma 89016, exposure time 15 ms, EM gain 15). Analyses were performed in ImageJ (Wayne Rasband, NIH) as described below. Each experiment was repeated at least three separate times with identical results.

Fluorescence imaging of RDP-2 with APT1/APT2 shRNA. shRNA plasmids were constructed in pLKO.1 vector[6] by restriction cloning adapting the protocol available on Addgene (https://www.addgene.org/tools/protocols/plko/). Three shRNA expression vectors were constructed for APT1 and two shRNAs were constructed for APT2 using the Broad Institute's Online Genetic Perturbation Platform database (http://portals.broadinstitute.org/gpp/public/). The sequences for each target are shown in Table S1. Sequencing was validated at the University of Chicago Comprehensive Cancer Center DNA Sequencing and Genotyping Facility. For experiments, the appropriate shRNA clones (50 ng/μL) were pooled. The pLKO.1 vector was used as a control shRNA in comparision to shRNAs for the targets of interest. 120,000 HEK293T cells/well were plated in 500 μL DMEM glutamax (10% FBS) into two wells of a four well chambered imaging dish (D35C4-20-1.5-N, Cellvis) which were precoated with 4 μg Poly-D-lysine (30-70 KDa, Alfa Aesar) for 2 h at room temperature or overnight at 4 ºC. After 18-20 h, the media was replaced by fresh 500 μL DMEM glutamax (10% FBS) and the cells were transfected with 600 ng control/APT1/APT2 shRNAs following manufacture's conditions. Briefly, 26.2 μL of opti-MEM containing 1.8 μL of Lipofectamine 3000 was added to mix of 13.6 μL opti-MEM, 1.2 μL P3000 and 12 μL shRNAs mix (50 ng/μL), and resulting DNA:Lipofectamine mix was incubated at room temperature for 15 min. After incubation 52 μL of the DNA:Lipofectamine mix was added to the corresponding well of the four well chambered imaging dish. After 32-35 h the media was replaced with 100 nM MitoTracker Deep red in 500 μL serum-free DMEM. After 30 min of incubation at 37 ºC, the cells were washed with 500 μL of Live Cell Imaging Solution and replaced by 1 μM **RDP-2** in Live Cell Imaging Solution. After incubation for 10 min at 37 °C and 5% CO_2, the cells were washed with 500 μL Live Cell Imaging Solution and 500 μL of Live Cell Imaging Solution and images were obtained on an inverted epi-fluorescence microscope (Olympus IX83) attached with EMCCD camera (Photometrics Evolve Delta) with 40x oil objective (N/A 1.3) for **RDP-2** (excitation filter 1 430/24 nm, dichroic chroma 89007, emission filter 1 470/24 nm, exposure time 60 ms, EM gain 30, excitation filter 2 480/20 nm, dichroic chroma 49014, emission filter 2 575/40 nm, exposure time 50 ms, EM gain 30), brightfield (exposure time 50 ms, EM gain 30), and MitoTracker Deep Red: (excitation filter 640/30 nm, emission filter 705/72 nm, dichroic chroma 89016, exposure

time 15 ms, EM gain 15). Analyses were performed in ImageJ (Wayne Rasband, NIH). Each experiment was repeated in at least three biological replicates with identical results.

Quantification and display of fluorescence images of RDP-1 and RDP-2 for cultured cells. The probe channel data were expressed as a ratio of the blue channel divided by the green channel and normalized to the average fluorescence intenstiy of the control conditions. To eliminate errors from autofluorescence, the images were thresholded using the bluer channel images at the same minimum level that included the maximum number of cells in focus (as determined visually by the mitocondrial dye) while limiting the signal from areas where cells are not present. The average value of all nonzero pixels from the blue channel:green channel ratio image were calculated in ImageJ for three different measurements from each experiment. The percent deprotected was estimated by imaging **RDP-1/2** in buffer (20 mM HEPES, 150 mM NaCl, 0.1% Triton X-100, pH 7.4, 0.1% DMSO) and for dye deprotected with 20 mM DTT in buffer (20 mM HEPES, 150 mM NaCl, 0.1% Triton X-100, pH 7.4, 0.1% DMSO) for 1 h at room temperature before aliquoted into individual tubes and stored at -80 ºC. A fresh aliquot of the solution was thawed for each experiment and diluted to 250 nM (**RDP-1**) or 500 nM (**RDP-2**) in buffer (20 mM HEPES, 150 mM NaCl, 0.1% Triton X-100, pH 7.4, 10 mM DTT) and allowed to equilibrate for 30 m before being imaged. The resulting images were divided in the same manner as above and the average ratio was taken from at least three images in a circle with a diameter of 150 pixels drawn at the center of the image. The ratios obtained from deprotected and protected dyes were interpreted to be the ratio at 100% and 0%, respectively, and fit by linear least squares. The resulting equation for the line was used to estimate the percent deacylation of the dye.

Epifluorescent imaging of RDP-2 with human colon organoids. At least 24 h before imaging experiments, cultured colon organoids were split using a 25G needle and 1 mL syringe, pelleted, and 50-100 organoids per well were re-plated in eight-well chambered slides (20 μL droplets/well; 250 μL organoid growth media; Cellvis) for treatment and imaging. 30 min before the addition of **RDP-2**, the cell culture media was removed and cells were washed with 300 μL of serum-free FluoroBrite DMEM (Gibco, High Glucose, Glutamax, no FBS). The media was then replaced with 300 μL of serum-free FluoroBrite DMEM containing 0.2% DMSO carrier as a control, Palm B, or ML348 incubated for 30 min at 37 °C and 5% CO_2. The media was removed, the cells were washed with 300 μL of Live Cell Imaging Solution (ThermoFisher), and 300 μL of 5 μM **RDP-2** along with the appropriate inhibitor or DMSO as control in Live Cell Imaging Solution was added to each well. After incubation for 10 min at 37 °C and 5% CO_2, the cells were washed with 300 μL Live Cell Imaging Solution and 300 μL of Live Cell Imaging

Solution containing DMSO or the appropriate inhibitor was added to the cells immediately before images were obtained on an inverted epi-fluorescence microscope (Olympus IX83) attached with EMCCD camera (Photometrics Evolve Delta) with 40x oil objective (N/A 1.3) for **RDP-2** (excitation filter 1 430/24 nm, dichroic chroma 89007, emission filter 1 470/24 nm, exposure time 150 ms, EM gain 100, excitation filter 2 480/20 nm, dichroic chroma 49014, emission filter 2 575/40 nm, exposure time 75 ms, EM gain 75), brightfield (exposure time 50 ms, EM gain 30). Analyses were performed in ImageJ (Wayne Rasband, NIH). Each experiment was repeated at least three separate times with identical results. Images were quantified and displayed in a similar maner to the cell culture experiments except that ROIs were manually selected for the tissue portion in focus with out setting a threshold.

Fluorescence imaging of RDP-1 with HEPG2 Cells. HEPG2 were plated with 500 mL of DMEM (Gibco, High Glucose, Glutamax, 10% FBS) a four well chambered imaging dish (D35C4-20-1.5-N, Cellvis) which were precoated with 4 μg Poly-D-lysine (30-70 KDa, Alfa Aesar) for 2 h at room temperature or overnight at 4 ºC and grown to *ca*. 80% confluence prior to imaging. 30 min before the addition of the RDP, the cell culture media was removed and cells were washed with 500 μL of serum-free DMEM (Gibco, High Glucose, Glutamax, no FBS). The media was then replaced with 500 μL of serum-free DMEM containing 100 nM Mitotracker Deep Red (ThermoFisher; to assist in focusing and processing) and either 0.1% DMSO carrier as a control or Palm B in DMSO and incubated for 30 min at 37 °C and 5% CO_2. The media was removed, the cells were washed with 500 μL of Live Cell Imaging Solution (ThermoFisher), and 500 μL of 1 μM **RDP-1** along with the Palm B or DMSO as control in Live Cell Imaging Solution was added to each well. After incubation for 10 min at 37 °C and 5% CO_2, the cells were washed with 500 μL Live Cell Imaging Solution and 500 μL of Live Cell Imaging Solution containing DMSO or the appropriate inhibitor was added to the cells immediately before images were obtained on an inverted epi-fluorescence microscope (Olympus IX83) attached with EMCCD camera (Photometrics Evolve Delta) with 40x oil objective (N/A 1.3; excitation filter 1 430/24 nm, dichroic chroma 89007, emission filter 1 470/24 nm, exposure time 80 ms, EM gain 60, excitation filter 2 480/20 nm, dichroic chroma 49014, emission filter 2 575/40 nm, exposure time 50 ms, EM gain 30), brightfield: (exposure time 50 ms, EM gain 30), and MitoTracker Deep Red: (excitation filter 640/30 nm, emission filter 705/72 nm, dichroic chroma 89016, exposure time 15 ms, EM gain 15). Analyses were performed in ImageJ (Wayne Rasband, NIH) as described above. Each experiment was repeated at least three separate times with identical results.

Preparation of palmitate solution. A stock solution of 5 mM sodium palmitate in 5% BSA

(fatty acid free, Sigma) in DMEM (Gibco, High Glucose, Glutamax, no FBS) was prepared by disolving BSA in DMEM and sodium palmitate was added. The resulting suspention was sonicated for 30 mins and then incubated at 37 ºC for 24 h. The now clear solution was then briefly mixed on a vortex mixter and filtered through a 0.22 μm syringe filter (PVDF, 30mm diameter, Santa Cruz Biotechnology, Dallas, TX). The resulting solution was alloquoted and stored at -20 ºC. Solutions containing only 5% BSA were prepared in the same way as a control. Solutions were thawed immediately before use and diluted to the required concentration in DMEM (Gibco, High Glucose, Glutamax, no FBS).

Fluorescence imaging of lipid metabolism in HEPG2 Cells with RDP-1. HEPG2 were plated with 500 mL of DMEM (Gibco, High Glucose, Glutamax, 10% FBS) a four well chambered imaging dish (D35C4-20-1.5-N, Cellvis) which were precoated with 4 μg Poly-D-lysine (30-70 KDa, Alfa Aesar) for 2 h at room temperature or overnight at 4 ºC and grown to *ca*. 70% confluence prior to serum starvation overnight (DMEM, Gibco, High Glucose, Glutamax, no FBS). 6 h before the experiment, media was replaced with DMEM (Gibco, High Glucose, Glutamax, no FBS) with 1% BSA (fatty acid free, Sigma) with 1 mM sodium palmitate or with out as a control. After 6 h, the cell culture media was removed and cells were washed with 500 μL of serum-free DMEM (Gibco, High Glucose, Glutamax, no FBS). The media was then replaced with 500 μL of serum-free DMEM containing 100 nM Mitotracker Deep Red (ThermoFisher; to assist in focusing and processing) and incubated for 30 min at 37 °C and 5% CO_2. The media was removed, the cells were washed with 500 μL of Live Cell Imaging Solution (ThermoFisher), and 500 μL of 1 μM **RDP-1** in Live Cell Imaging Solution was added to each well. After incubation for 10 min at 37 °C and 5% CO_2, the cells were washed with 500 μL Live Cell Imaging Solution and 500 μL of Live Cell Imaging Solution containing DMSO or the appropriate inhibitor was added to the cells immediately before images were obtained on an inverted epi-fluorescence microscope (Olympus IX83) attached with EMCCD camera (Photometrics Evolve Delta) with 40x oil objective (N/A 1.3; excitation filter 1 430/24 nm, dichroic chroma 89007, emission filter 1 470/24 nm, exposure time 80 ms, EM gain 60, excitation filter 2 480/20 nm, dichroic chroma 49014, emission filter 2 575/40 nm, exposure time 50 ms, EM gain 30). Analyses were performed in ImageJ (Wayne Rasband, NIH) as described above. Each experiment was repeated at least three separate times with identical results.

1. R. S. Kathayat, P. D. Elvira and B. C. Dickinson, *Nat Chem Biol*, 2017, **13**, 150-152.
2. M. R. Vippila, P. K. Ly and G. D. Cuny, *J Nat Prod*, 2015, **78**, 2398-2404.
3. D. R. Rooker and D. Buccella, *Chem Sci*, 2015, **6**, 6456-6461.

4. S. Y. Park, M. Ebihara, Y. Kubota, K. Funabiki and M. Matsui, *Dyes Pigments*, 2009, **82**, 258-267.
5. T. Sato, D. E. Stange, M. Ferrante, R. G. Vries, J. H. Van Es, S. Van den Brink, W. J. Van Houdt, A. Pronk, J. Van Gorp, P. D. Siersema and H. Clevers, *Gastroenterology*, 2011, **141**, 1762-1772.
6. J. Moffat, D. A. Grueneberg, X. Yang, S. Y. Kim, A. M. Kloepfer, G. Hinkle, B. Piqani, T. M. Eisenhaure, B. Luo, J. K. Grenier, A. E. Carpenter, S. Y. Foo, S. A. Stewart, B. R. Stockwell, N. Hacohen, W. C. Hahn, E. S. Lander, D. M. Sabatini and D. E. Root, *Cell*, 2006, **124**, 1283-1298.

Table S1 | Sequences of RNAi used in this work.

Target	Sequence
LYPLA1 (APT1)	CGGTGGTGCTAATAGAGATAT
LYPLA1 (APT1)	CTATGCCTTCATGGTTTGATA
LYPLA1 (APT1)	CAGGAAATGATGGATGTCAA
LYPLA2 (APT2)	GGCTGCTTTCTTATCCATTTC
LYPLA2 (APT2)	GCAGCTGTGAAGGAATTTCTT

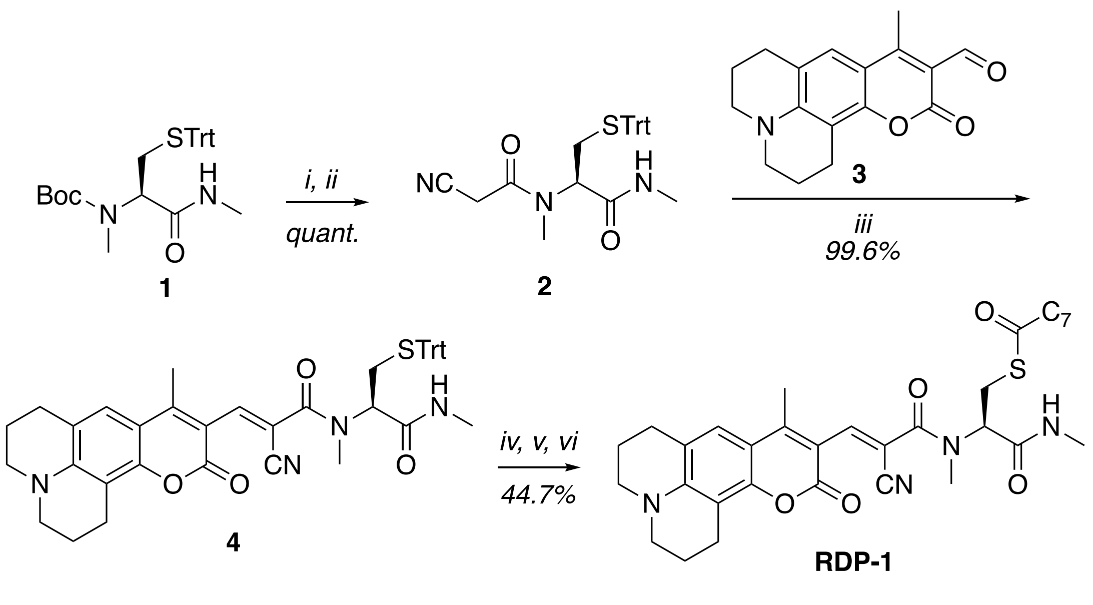

Figure S1 | Synthesis of Ratiometric Depalmitoylation Probe 1 (RDP-1). *i*. 20% TFA, DCM, N_2, 1 h *ii*. cyanoacetic acid, EDC•HCl, HoBt, DIPEA, DMF, 0 ºC to rt, 12 h. *iii*. Piperdine, EtOH:DCM 1:9, 72 h i*v*. I_2, MeOH, 30 min *v*. DTT, DIPEA, DMF *vi*. Octanoic anhydride, DIPEA, DMF.

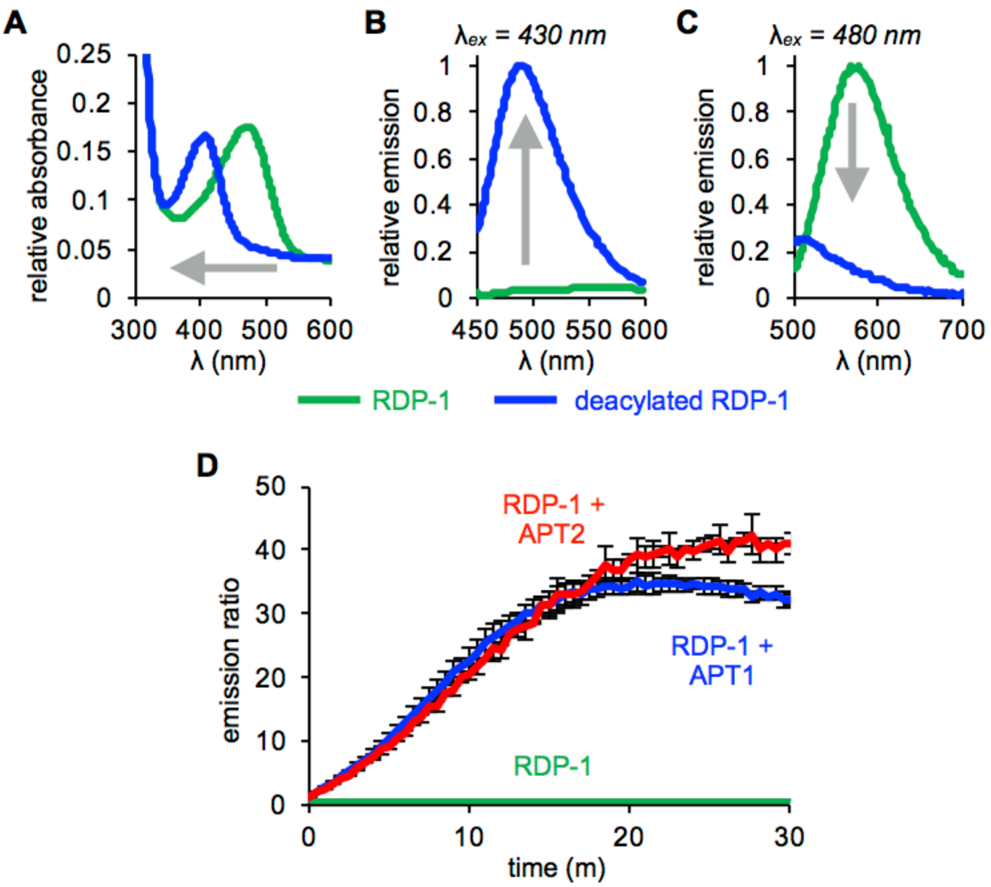

Figure S2 | Photophysical properties and deacylation response of RDP-1. UV-Vis absorption (A) and fluorescence emission at λ_{ex} = 430 nm (B) and λ_{ex} = 480 nm (C) of 15 μM **RDP-1** (green) and deacylated **RDP-1** (blue) in buffer (20 mM HEPES, 150 mM NaCl, 0.1% Triton X-100, pH 7.4). (D) Kinetic assay of 1 μM **RDP-1** with 200 nM APT1 or 200 nM APT2. Error bars are ± std. dev.

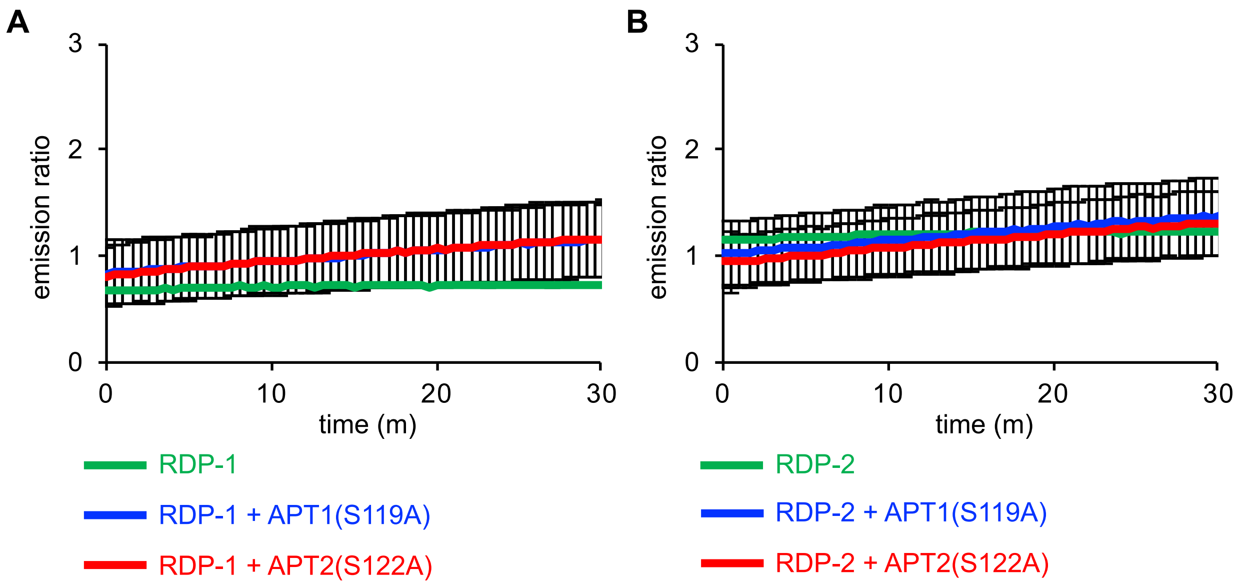

Figure S3 | Response of RDP-1 and RDP-2 to catalytically inactive APT1 and APT2 *in vitro*. Kinetic assay of 1 μM (A) **RDP-1** and (B) **RDP-2** with 2 μM APT1(S119A) (blue), 2 μM APT2(S122A) (red), and no enzyme (green) in buffer (20 mM HEPES, 150 mM NaCl, 0.1% Triton X-100, pH 7.4). Error bars are ± standard deviation (n = 3).

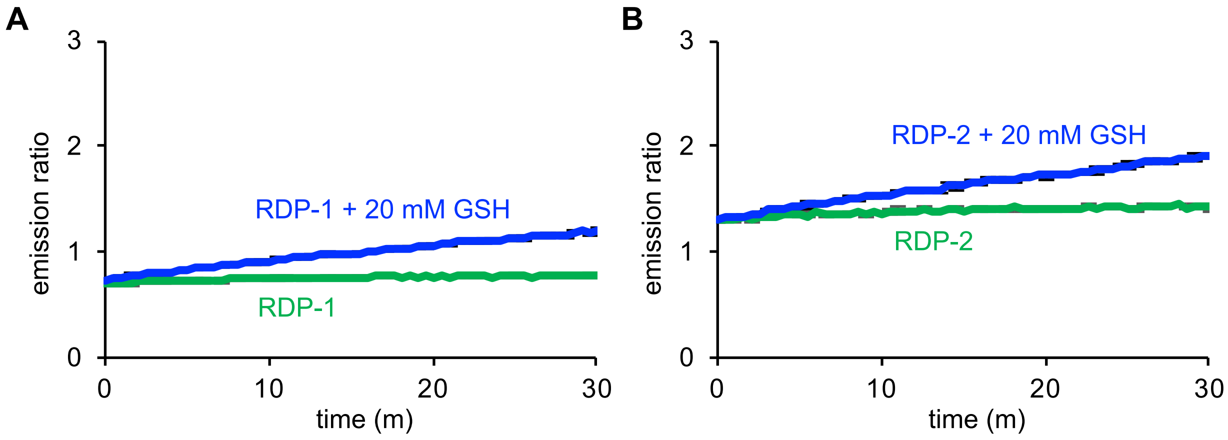

Figure S4 | Response of RDP-1 and RDP-2 to glutathione *in vitro*. Kinetic assay of 1 μM (A) **RDP-1** and (B) **RDP-2** with 20 mM glutathione (blue) or without (green) in buffer (20 mM HEPES, 150 mM NaCl, 0.1% Triton X-100, pH 7.4). Error bars are ± standard deviation (n = 3).

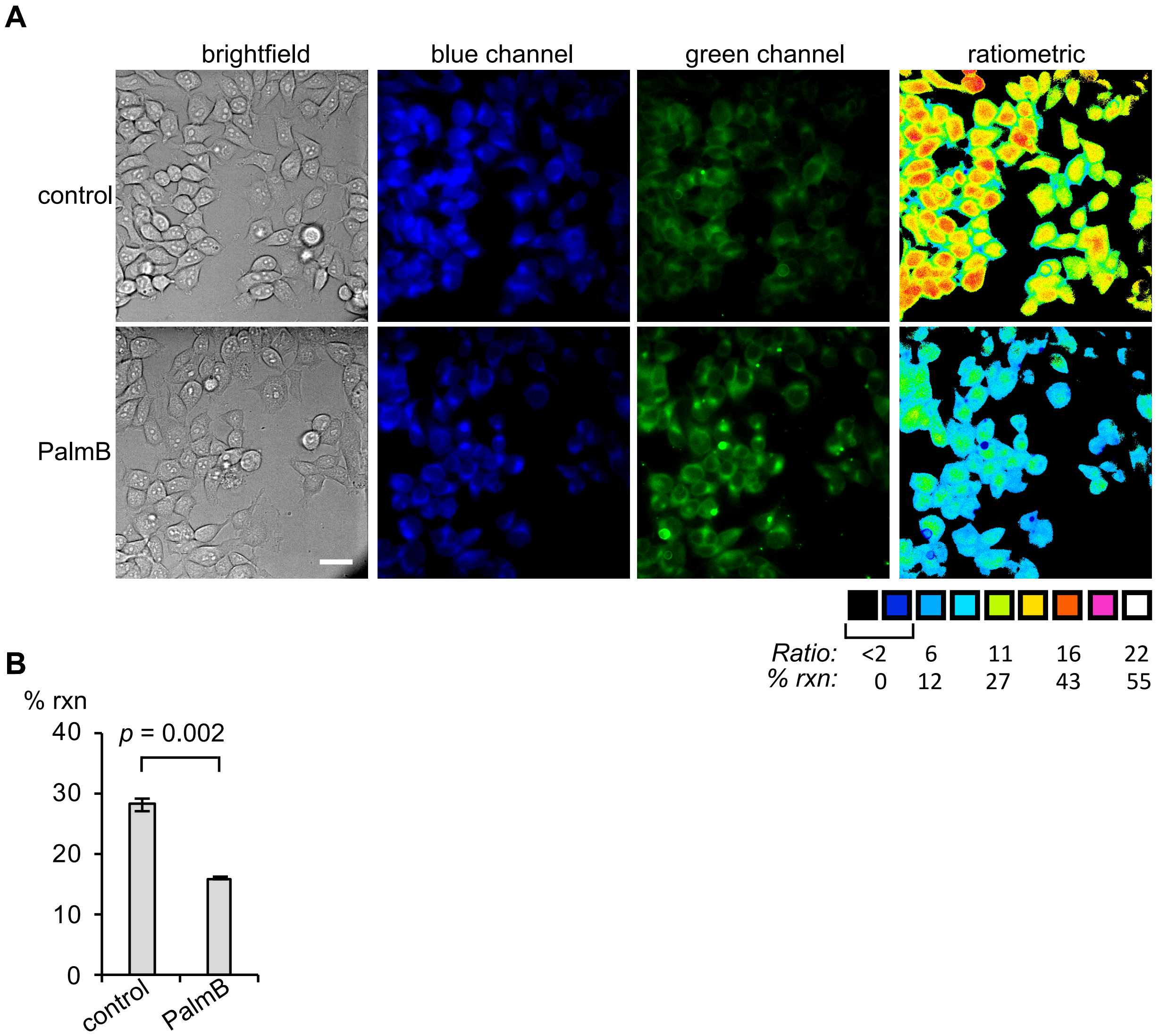

Figure S5 | Response of RDP-1 to PalmB inhibition in HEK293T cells. (A) HEK293T cells loaded with 1 μM **RDP-1** for 10 min after treatment with either DMSO or 20 μM PalmB for 30 min and then analyzed by fluorescence microscopy. A representative set of images from each condition are shown, including brightfield, "blue" fluorescence (λ_{ex} = 430 nm, λ_{em} = 470 nm), "green" fluorescence (λ_{ex} = 480 nm, λ_{em} = 575 nm), and the ratiometric images generated by dividing the blue channel by the green channel. (B) Quantification of ratiometric response of experiment described in (A). Scale bar = 20 μm. Error bars are ± standard deviation (n = 3).

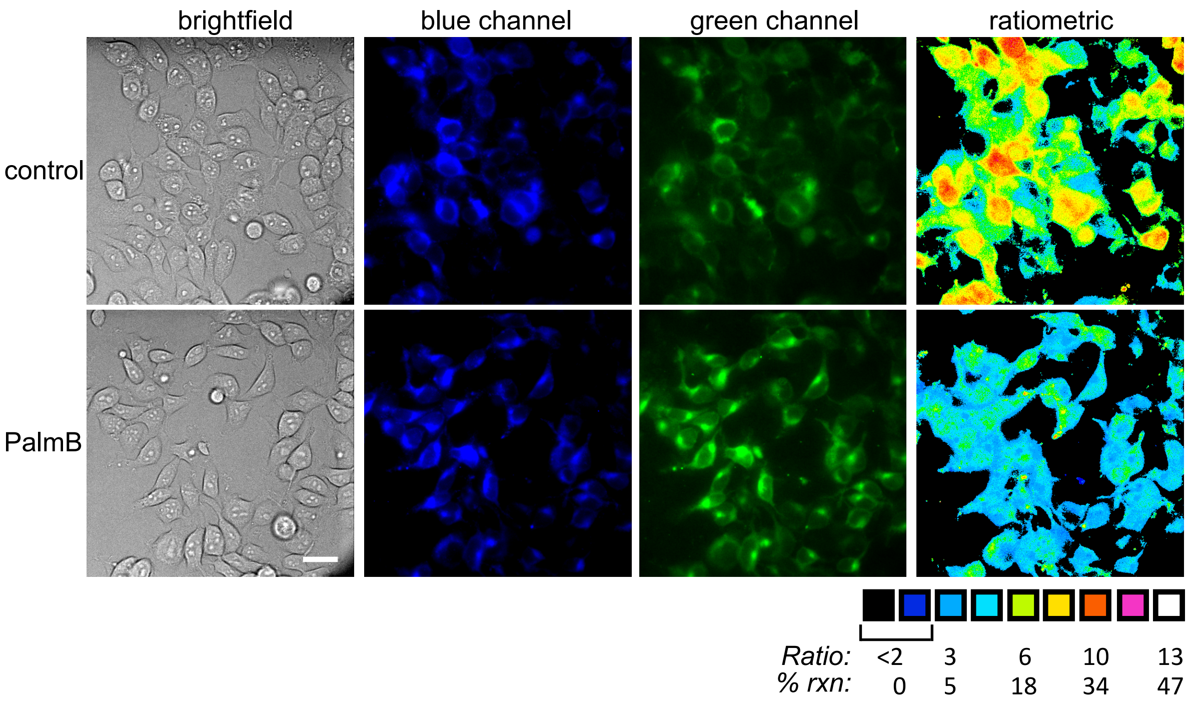

Figure S6 | Response of RDP-2 to PalmB inhibition in HEK293T cells (full set of images from Fig. 2). HEK293T cells loaded with 1 μM **RDP-2** for 10 min after treatment with either DMSO or 20 μM PalmB for 30 min and then analyzed by fluorescence microscopy. A representative set of images from each condition are shown, including brightfield, "blue" fluorescence (λ_{ex} = 430 nm, λ_{em} = 470 nm), "green" fluorescence (λ_{ex} = 480 nm, λ_{em} = 575 nm), and the ratiometric images generated by dividing the blue channel by the green channel. Scale bar = 20 μm.

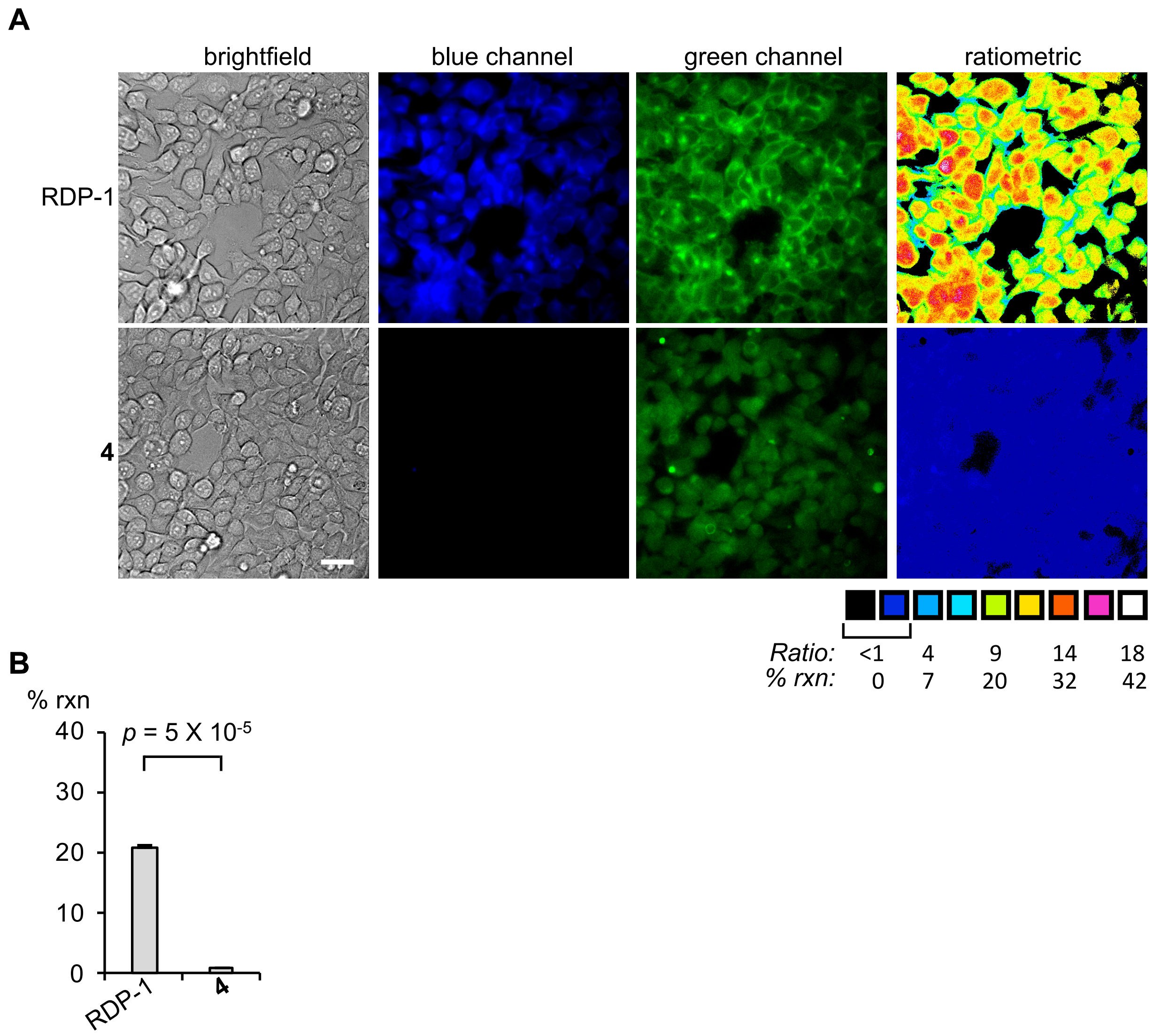

Figure S7 | Response of RDP-1 and compound 4 in HEK293T cells. (A) HEK293T cells loaded with 1 μM **RDP-1** or **4** for 10 min and then analyzed by fluorescence microscopy. A representative set of images from each condition are shown, including brightfield, "blue" fluorescence (λ_{ex} = 430 nm, λ_{em} = 470 nm), "green" fluorescence (λ_{ex} = 480 nm, λ_{em} = 575 nm), and the ratiometric images generated by dividing the blue channel by the green channel. (B) Quantification of ratiometric response of experiment described in (A). Scale bar = 20 μm. Error bars are ± standard deviation (n = 3).

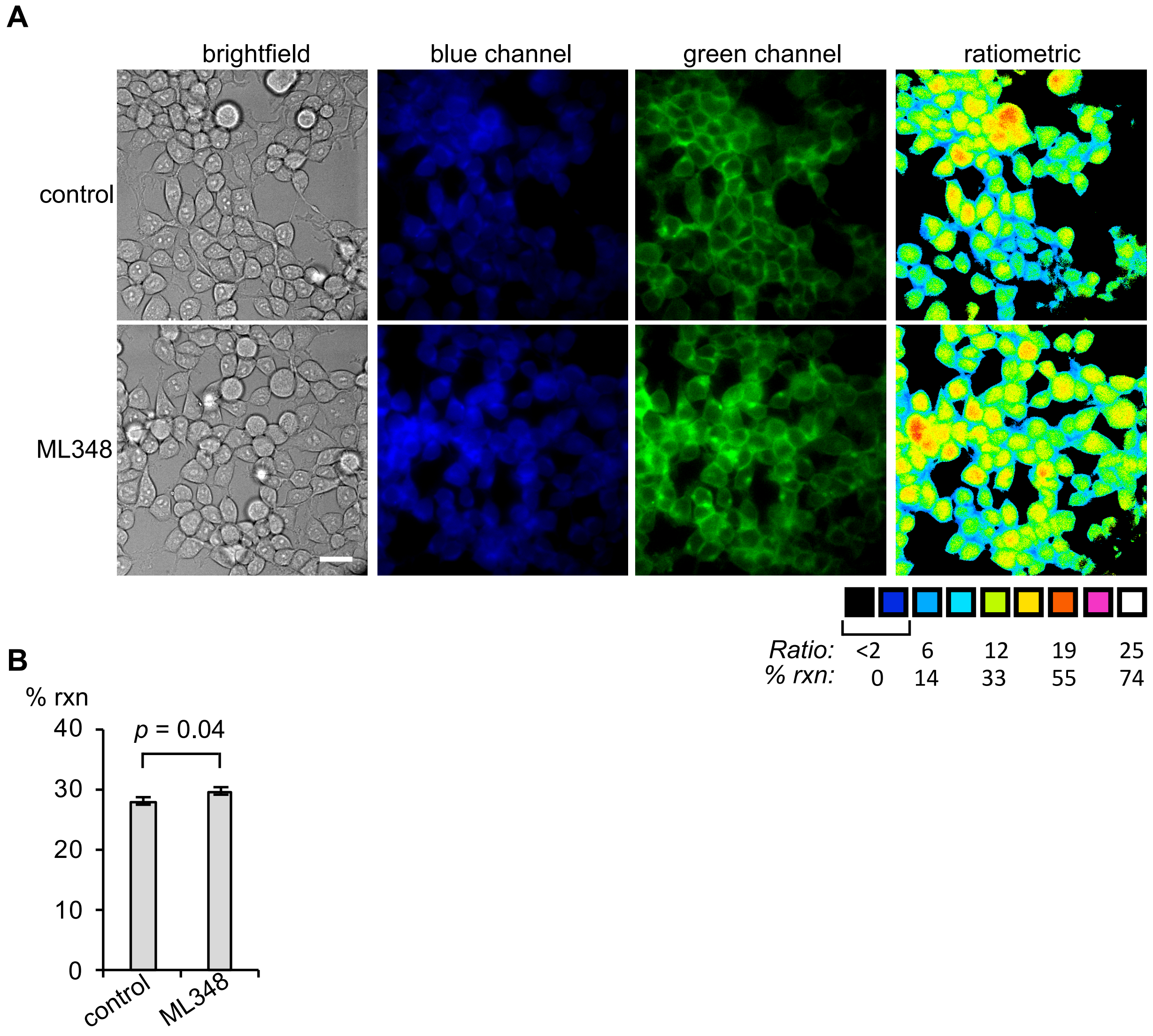

Figure S8 | Response of RDP-1 to ML348 inhibition in HEK293T cells. (A) HEK293T cells loaded with 1 μM **RDP-1** for 10 min after treatment with either DMSO or 10 μM ML348 for 30 min and then analyzed by fluorescence microscopy. A representative set of images from each condition are shown, including brightfield, "blue" fluorescence (λ_{ex} = 430 nm, λ_{em} = 470 nm), "green" fluorescence (λ_{ex} = 480 nm, λ_{em} = 575 nm), and the ratiometric images generated by dividing the blue channel by the green channel. (B) Quantification of ratiometric response of experiment described in (A). Scale bar = 20 μm. Error bars are ± standard deviation (n = 3).

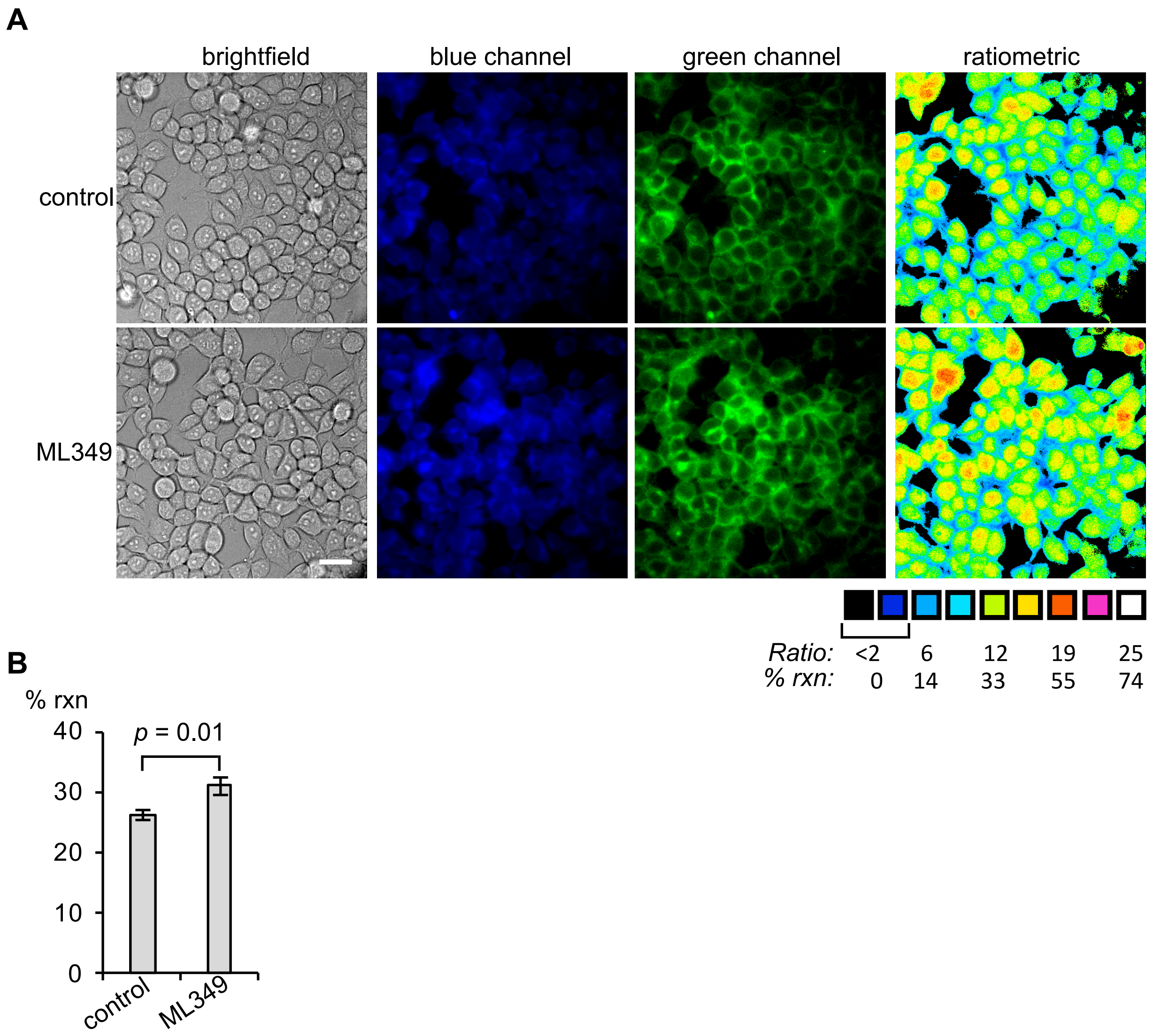

Figure S9 | Response of RDP-1 to ML349 inhibition in HEK293T cells. (A) HEK293T cells loaded with 1 μM **RDP-1** for 10 min after treatment with either DMSO or 10 μM ML349 for 30 min and then analyzed by fluorescence microscopy. A representative set of images from each condition are shown, including brightfield, "blue" fluorescence (λ_{ex} = 430 nm, λ_{em} = 470 nm), "green" fluorescence (λ_{ex} = 480 nm, λ_{em} = 575 nm), and the ratiometric images generated by dividing the blue channel by the green channel. (B) Quantification of ratiometric response of experiment described in (A). Scale bar = 20 μm. Error bars are ± standard deviation (n = 3).

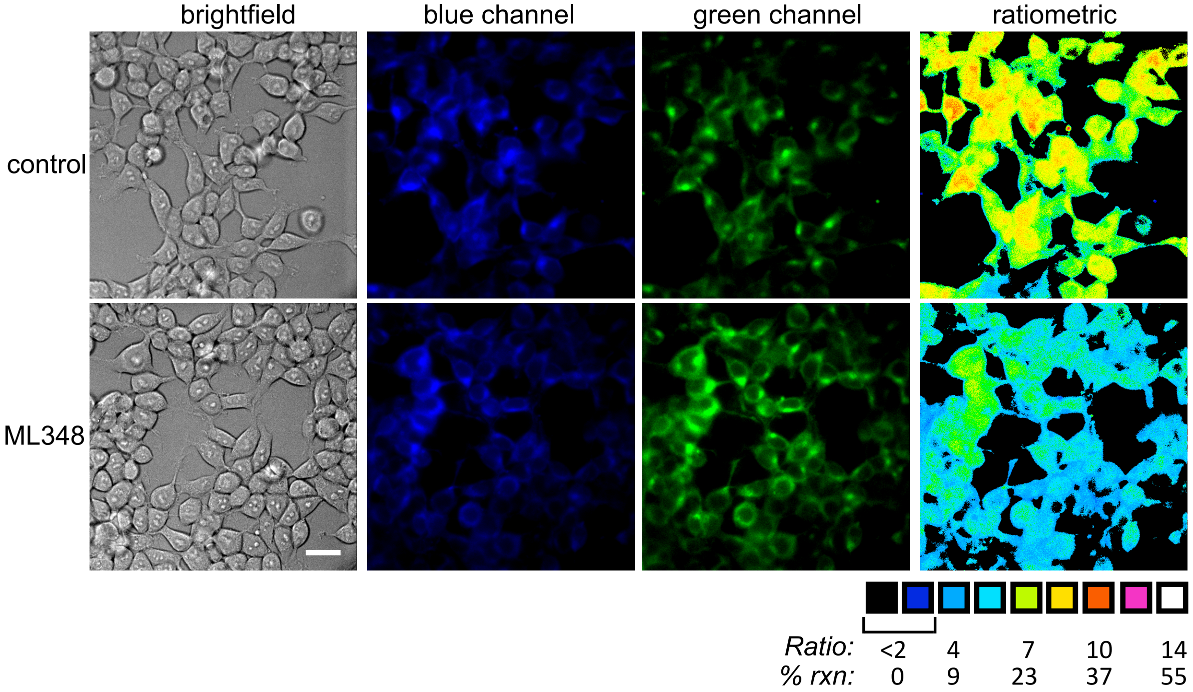

Figure S10 | Response of RDP-2 to ML348 inhibition in HEK293T cells (full set of images from Fig. 3). HEK293T cells loaded with 1 μM **RDP-2** for 10 min after treatment with either DMSO or 5 μM ML348 for 30 min and then analyzed by fluorescence microscopy. A representative set of images from each condition are shown, including brightfield, "blue" fluorescence (λ_{ex} = 430 nm, λ_{em} = 470 nm), "green" fluorescence (λ_{ex} = 480 nm, λ_{em} = 575 nm), and the ratiometric images generated by dividing the blue channel by the green channel. Scale bar = 20 μm.

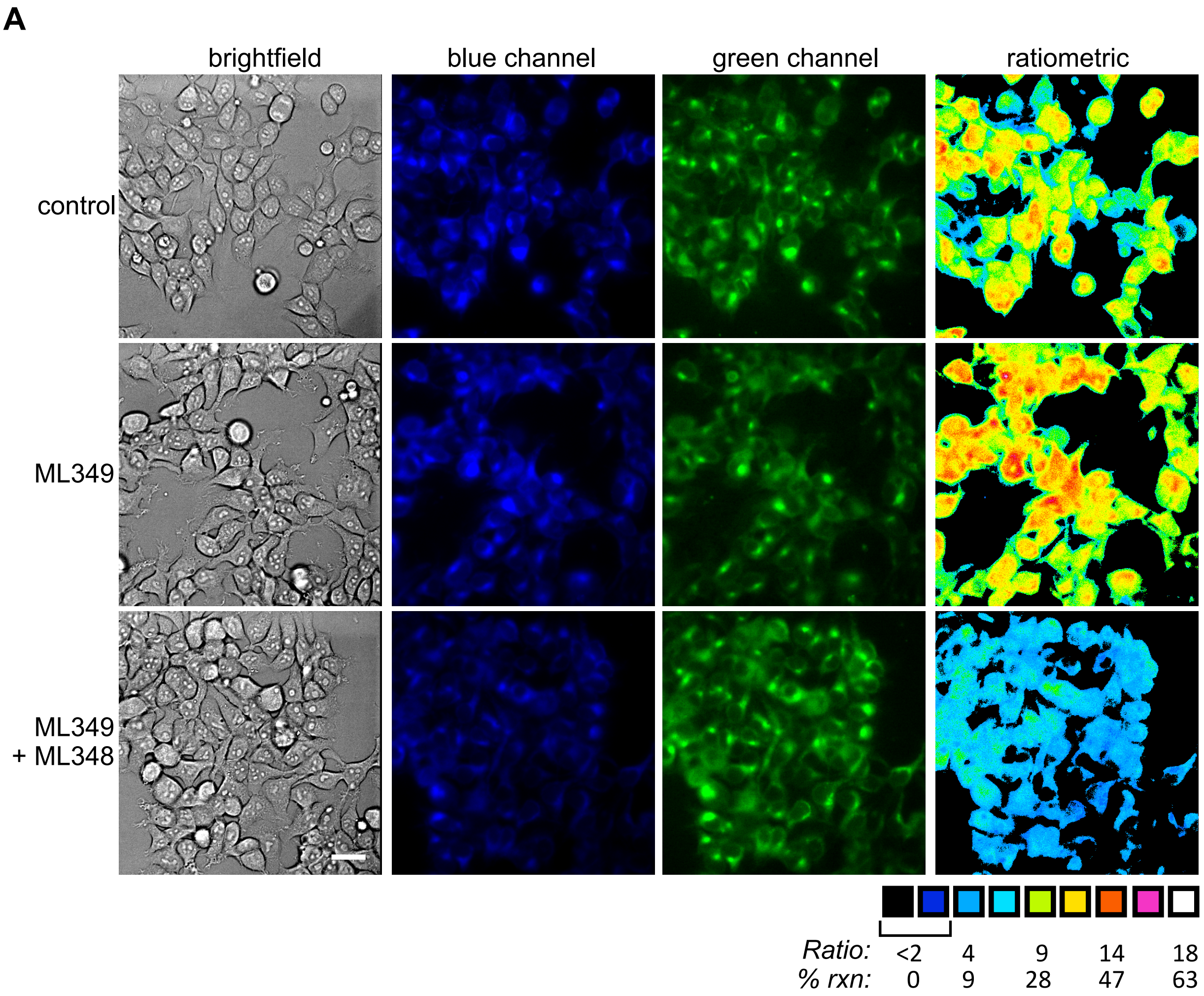

Figure S11 | Response of RDP-2 to ML349 inhibition in HEK293T cells (full set of images from Fig. 3). HEK293T cells loaded with 1 μM **RDP-2** for 10 min after treatment with either DMSO or 5 μM ML349 or 5 μM of ML349 and ML348 for 30 min and then analyzed by fluorescence microscopy. A representative set of images from each condition are shown, including brightfield, "blue" fluorescence (λ_{ex} = 430 nm, λ_{em} = 470 nm), "green" fluorescence (λ_{ex} = 480 nm, λ_{em} = 575 nm), and the ratiometric images generated by dividing the blue channel by the green channel. Scale bar = 20 μm.

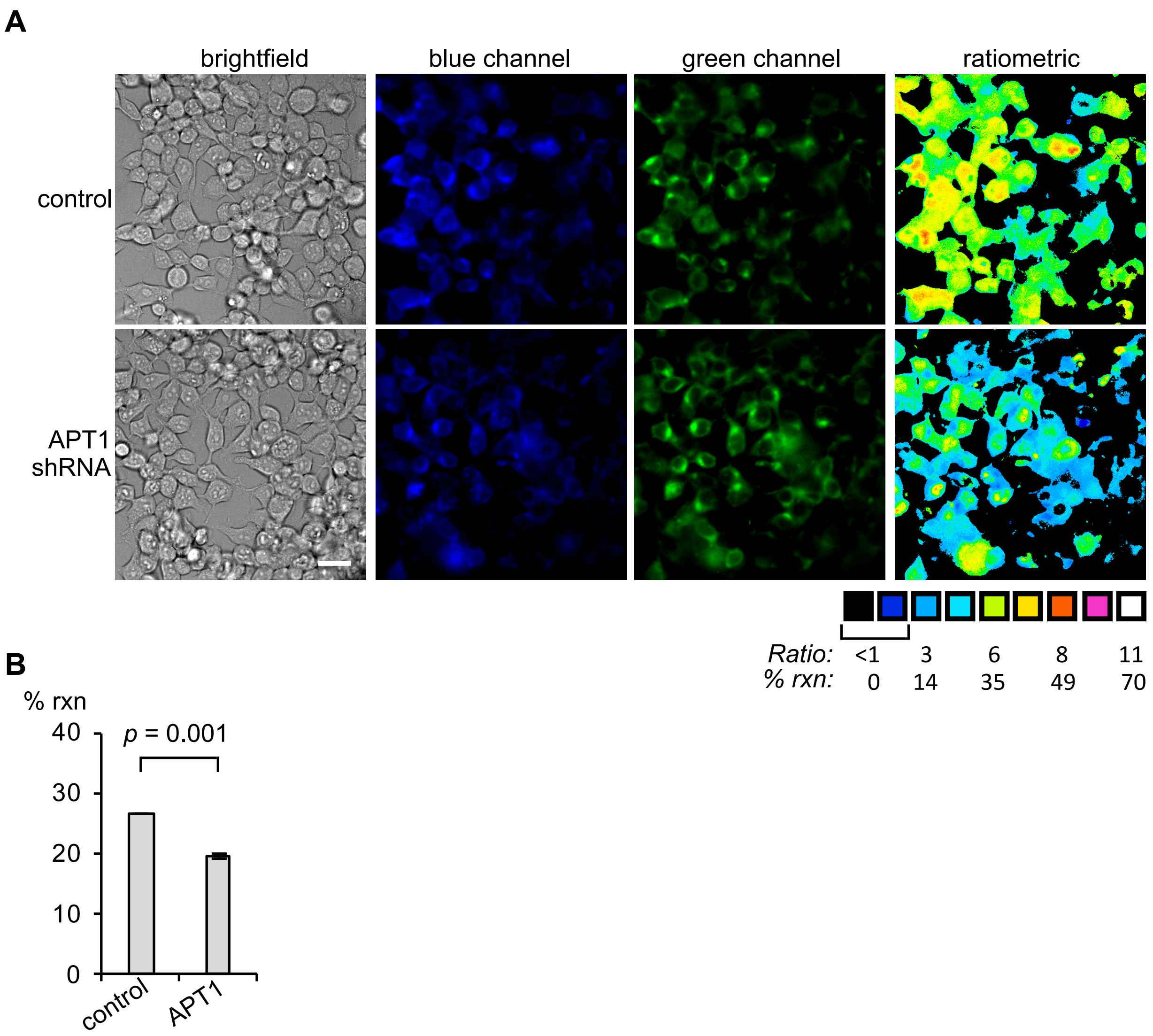

Figure S12 | Response of RDP-2 to APT1 knockdown in HEK293T cells. (A) HEK293T cells transfected with either control shRNA vector or a vector generating shRNA targeting APT1 for 48 hours, and then loaded with 1 μM **RDP-2** for 10 min and analyzed by fluorescence microscopy. A representative set of images from each condition are shown, including brightfield, "blue" fluorescence (λ_{ex} = 430 nm, λ_{em} = 470 nm), "green" fluorescence (λ_{ex} = 480 nm, λ_{em} = 575 nm), and the ratiometric images generated by dividing the blue channel by the green channel. (B) Quantification of ratiometric response of experiment described in (A). Scale bar = 20 μm. Error bars are ± standard deviation (n = 3).

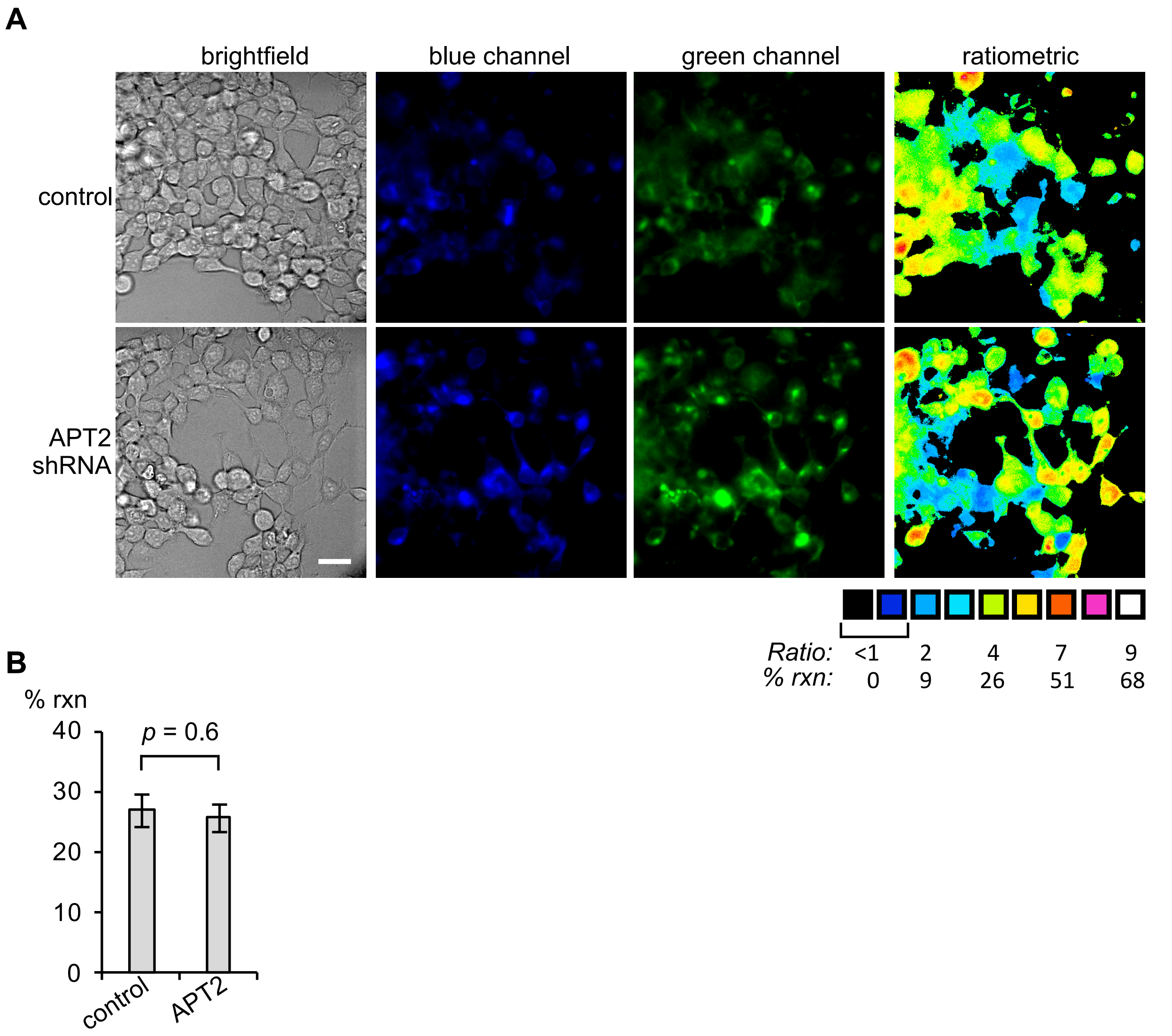

Figure S13 | Response of RDP-2 to APT2 knockdown in HEK293T cells. (A) HEK293T cells transfected with either control shRNA vector or a vector generating shRNA targeting APT2 for 48 hours, and then loaded with 1 μM **RDP-2** for x min and analyzed by fluorescence microscopy. A representative set of images from each condition are shown, including brightfield, "blue" fluorescence (λ_{ex} = 430 nm, λ_{em} = 470 nm), "green" fluorescence (λ_{ex} = 480 nm, λ_{em} = 575 nm), and the ratiometric images generated by dividing the blue channel by the green channel. (B) Quantification of ratiometric response of experiment described in (A). Scale bar = 20 μm. Error bars are ± standard deviation (n = 3).

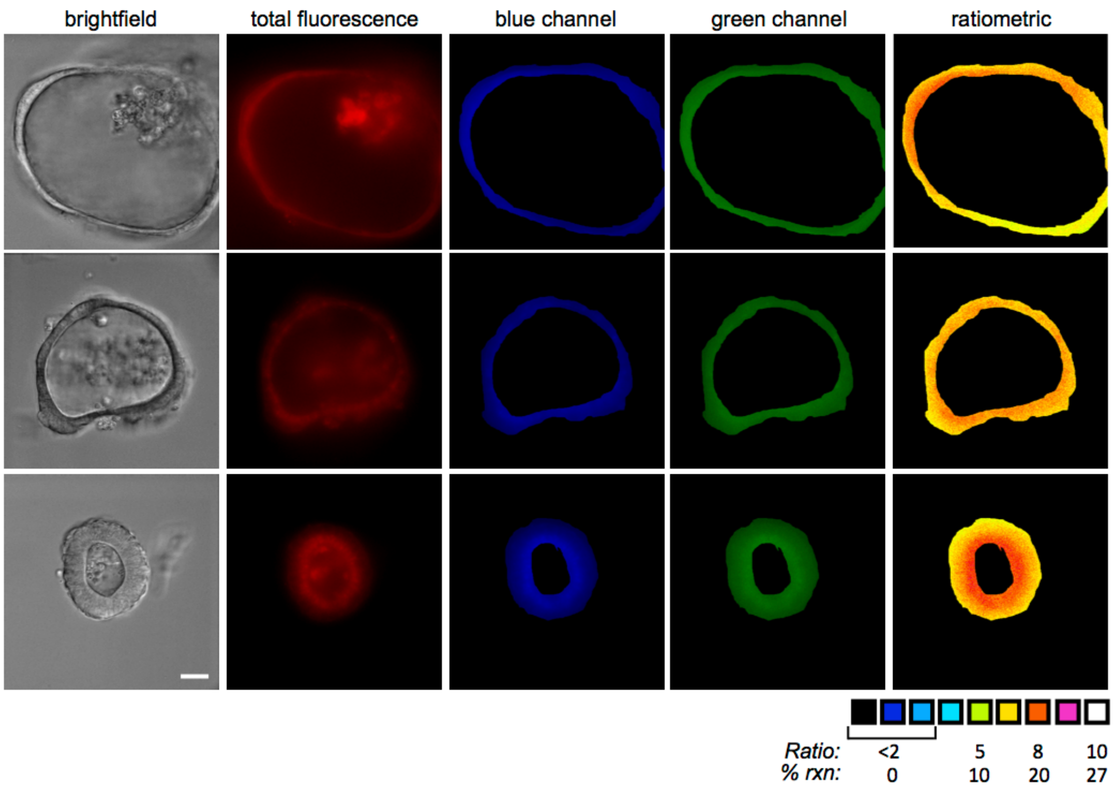

Figure S14 | Response of RDP-2 in colon organoids: DMSO-treated samples (full set of images from Fig. 4). (A) Colon cells organoids with 5 μM **RDP-2** for 10 min after treatment with DMSO for 30 min and then analyzed by fluorescence microscopy. A representative set of images from each condition are shown, including brightfield, total fluorescence of "blue" + "green" fluorescence, "blue" fluorescence (λ_{ex} = 430 nm, λ_{em} = 470 nm), "green" fluorescence (λ_{ex} = 480 nm, λ_{em} = 575 nm), and the ratiometric images generated by dividing the blue channel by the green channel. Scale bar = 20 μm.

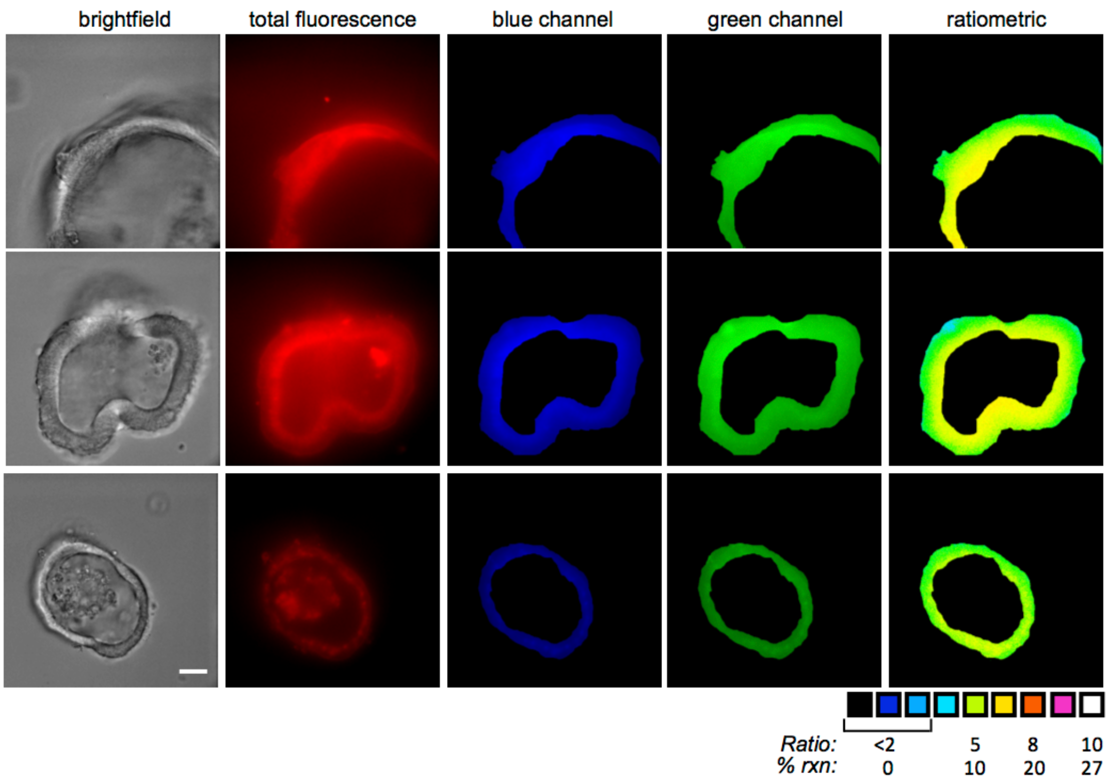

Figure S15 | Response of RDP-2 to PalmB inhibition in colon organoids: PalmB-treated samples (full set of images from Fig. 4). (A) Colon organoids loaded with 5 μM **RDP-2** for 10 min after treatment with 40 μM PalmB for 30 min and then analyzed by fluorescence microscopy. A representative set of images from each condition are shown, including brightfield, total fluorescence of "blue" + "green" fluorescence, "blue" fluorescence (λ_{ex} = 430 nm, λ_{em} = 470 nm), "green" fluorescence (λ_{ex} = 480 nm, λ_{em} = 575 nm), and the ratiometric images generated by dividing the blue channel by the green channel. Scale bar = 20 μm.

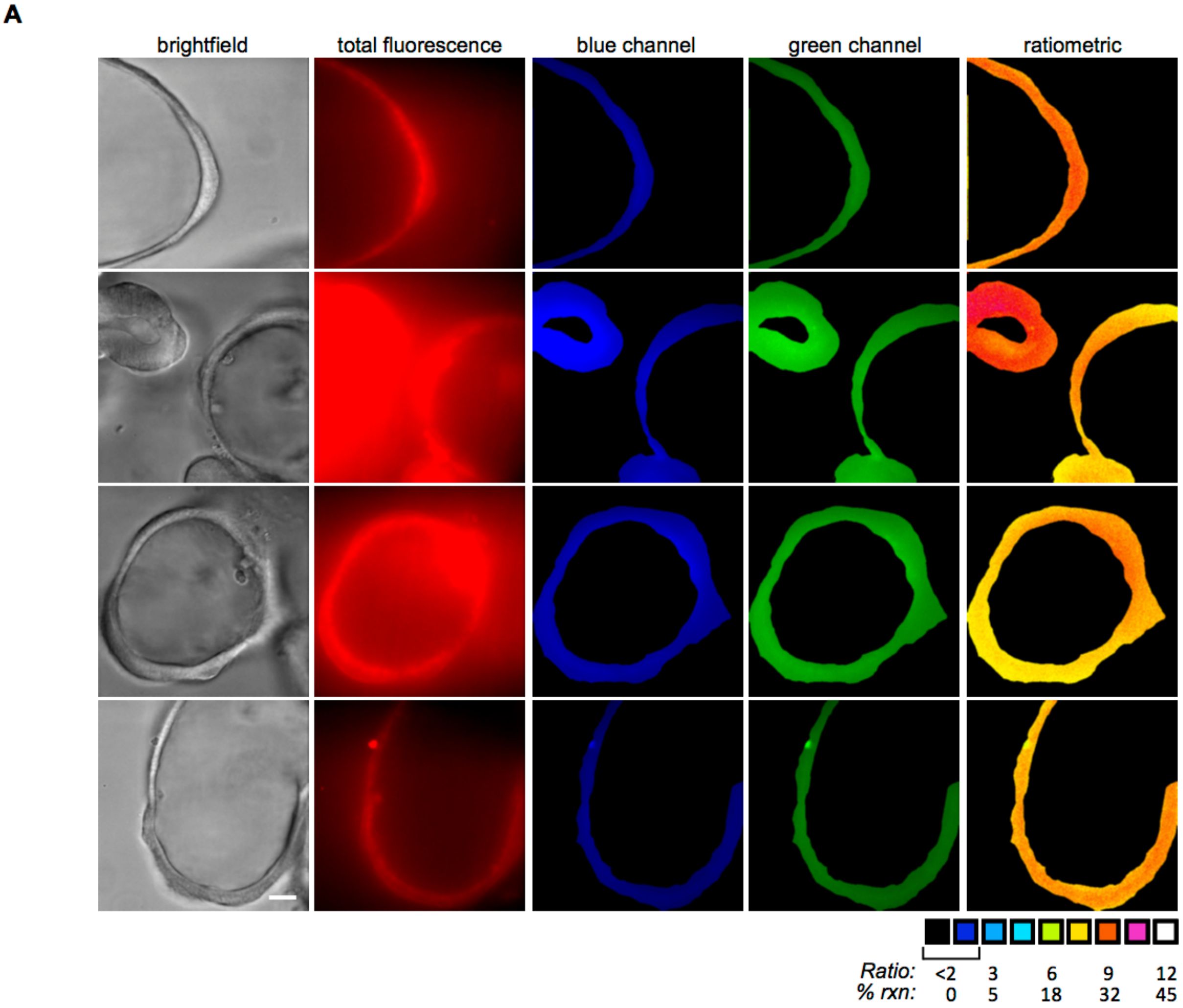

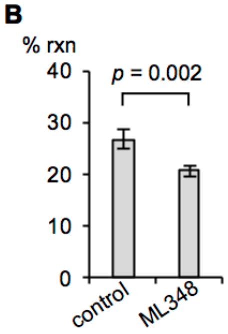

Figure S16 | Response of RDP-2 to ML348 inhibition in colon organoids: DMSO-treated samples. (A) Colon organoids loaded with 5 μM **RDP-2** for 10 min after treatment with DMSO for 30 min and then analyzed by fluorescence microscopy. A representative set of images from each condition are shown, including brightfield, total fluorescence of "blue" + "green" fluorescence, "blue" fluorescence (λ_{ex} = 430 nm, λ_{em} = 470 nm), "green" fluorescence (λ_{ex} = 480

nm, λ_{em} = 575 nm), and the ratiometric images generated by dividing the blue channel by the green channel. (B) Quantification of ratiometric response of experiment described in (A). Scale bar = 20 μm. Error bars are ± standard deviation (n = 4).

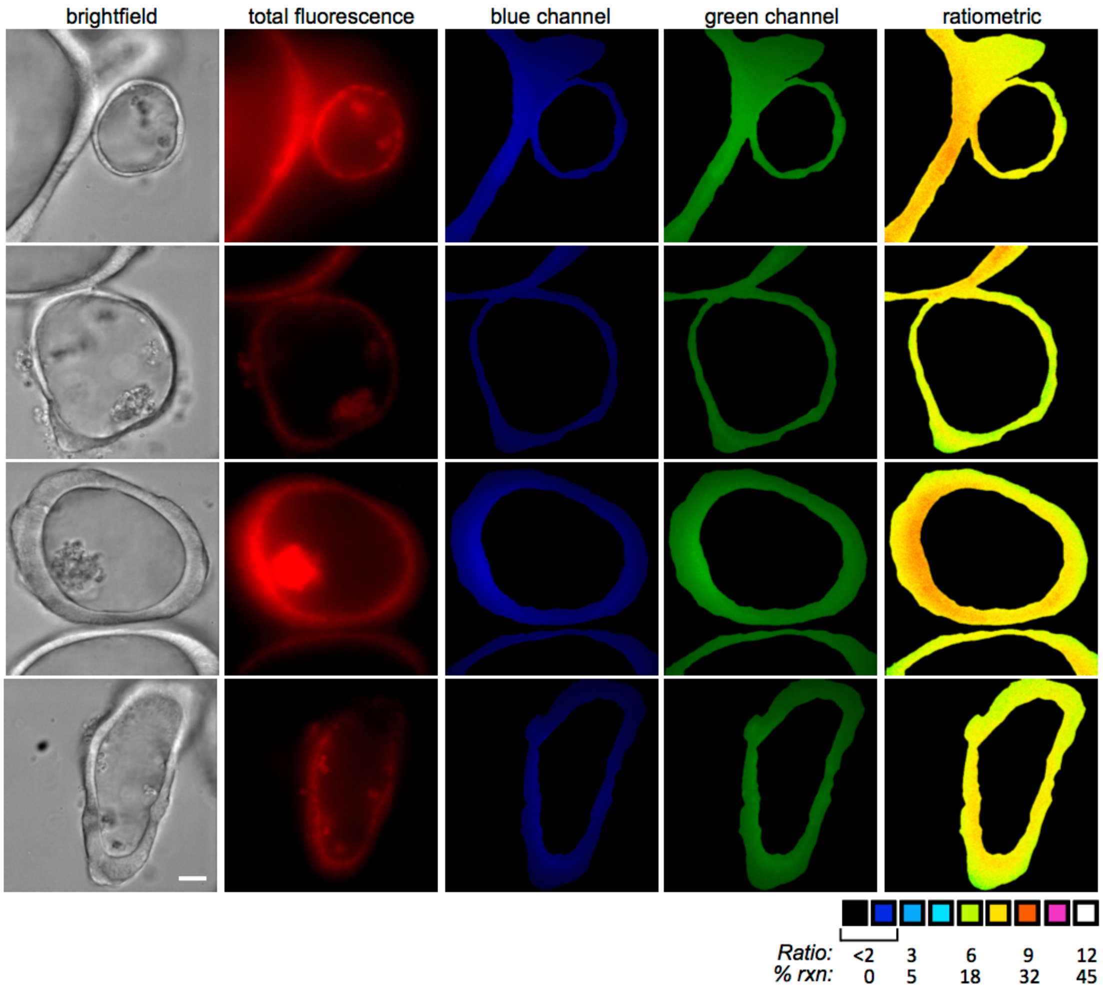

Figure S17 | Response of RDP-2 to ML348 inhibition in colon organoids: ML348-treated samples. (A) colon organoids loaded with 5 μM **RDP-2** for 10 min after treatment with 40 μM ML348 for 30 min and then analyzed by fluorescence microscopy. A representative set of images from each condition are shown, including brightfield, total fluorescence of "blue" + "green" fluorescence, "blue" fluorescence (λ_{ex} = 430 nm, λ_{em} = 470 nm), "green" fluorescence (λ_{ex} = 480 nm, λ_{em} = 575 nm), and the ratiometric images generated by dividing the blue channel by the green channel. Scale bar = 20 μm.

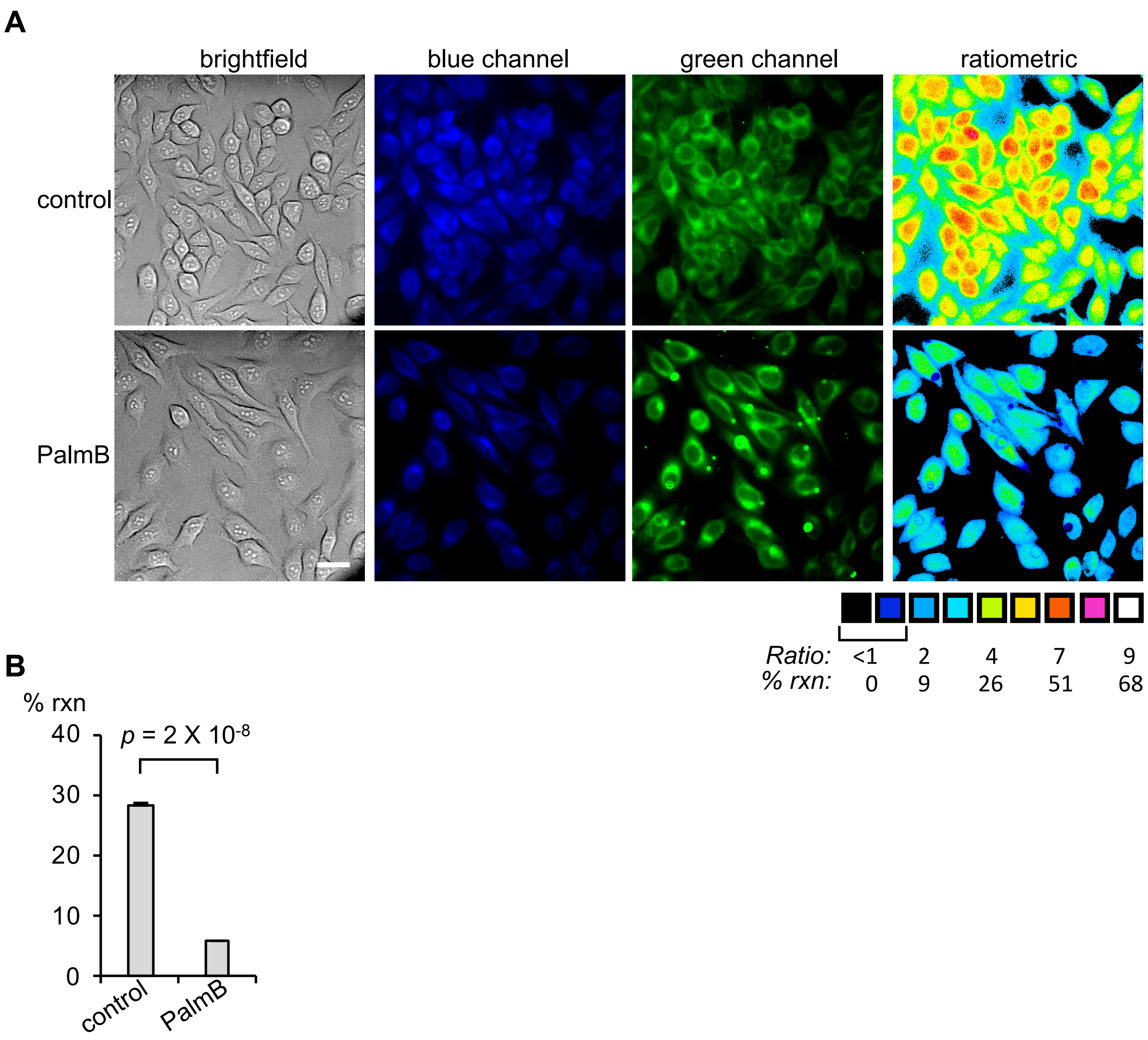

Figure S18 | Response of RDP-1 to PalmB inhibition in HEPG2 cells. (A) HEPG2 cells loaded with 1 μM **RDP-1** for 10 min after treatment with either DMSO or 40 μM PalmB for 30 min and then analyzed by fluorescence microscopy. A representative set of images from each condition are shown, including brightfield, "blue" fluorescence (λ_{ex} = 430 nm, λ_{em} = 470 nm), "green" fluorescence (λ_{ex} = 480 nm, λ_{em} = 575 nm), and the ratiometric images generated by dividing the blue channel by the green channel. (B) Quantification of ratiometric response of experiment described in (A). Scale bar = 20 μm. Error bars are ± standard deviation (n = 3).

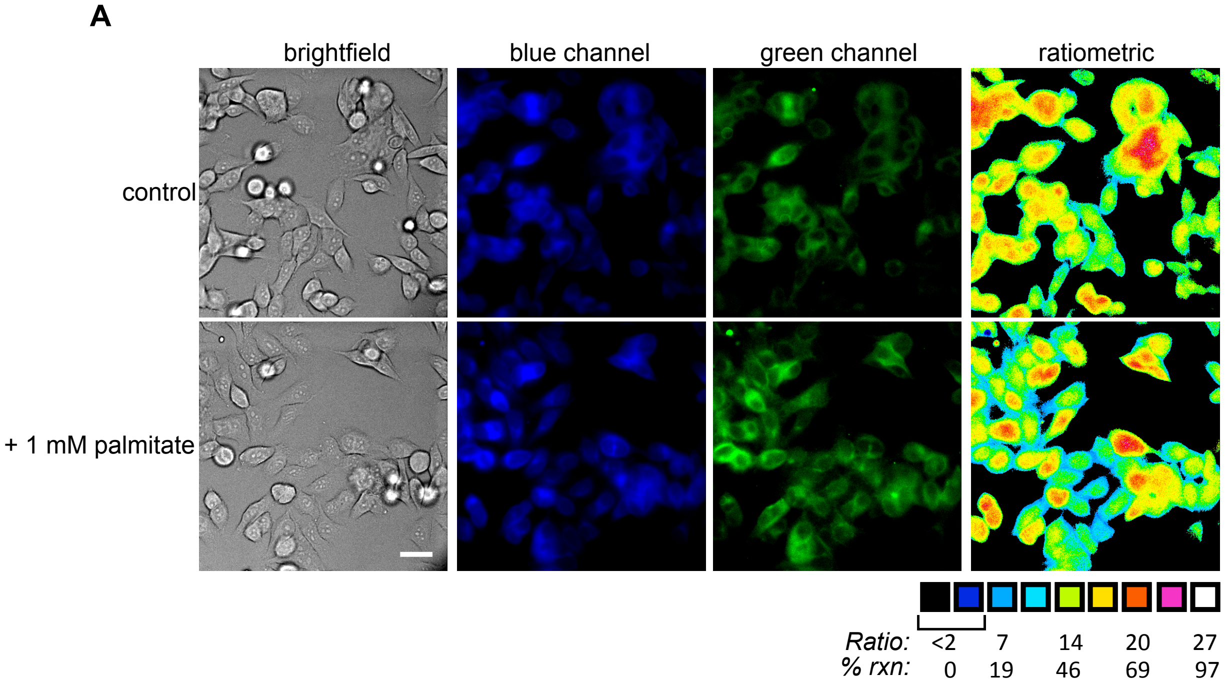

Figure S19 | Response of RDP-1 to palmitate in HEPG2 cells (full set of images from Fig. 5). (A) Serum-starved HEPG2 cells loaded with 1 μM **RDP-1** for 10 min after treatment with either 1% BSA or 1 mM palmitate in 1% BSA for 6 h and then analyzed by fluorescence microscopy. A representative set of images from each condition are shown, including brightfield, "blue" fluorescence (λ_{ex} = 430 nm, λ_{em} = 470 nm), "green" fluorescence (λ_{ex} = 480 nm, λ_{em} = 575 nm), and the ratiometric images generated by dividing the blue channel by the green channel. Scale bar = 20 μm.

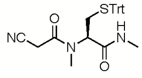

2

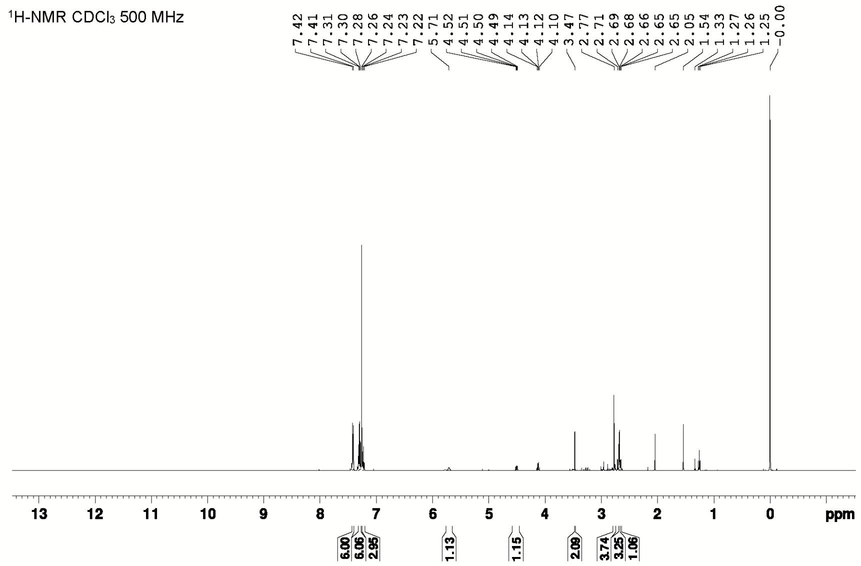

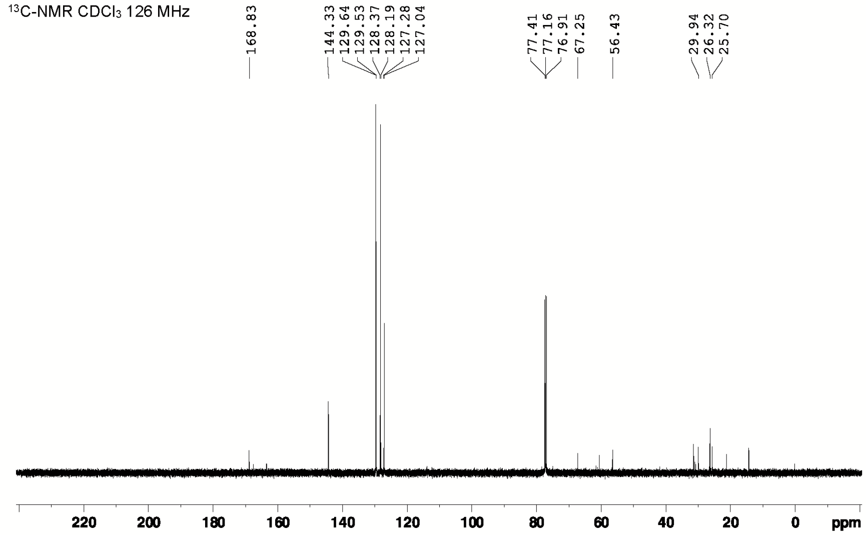

^{1}H NMR and $^{13-}$NMR of 2.

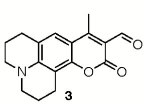

3

^{1}H-NMR $CDCl_3$ 500 MHz

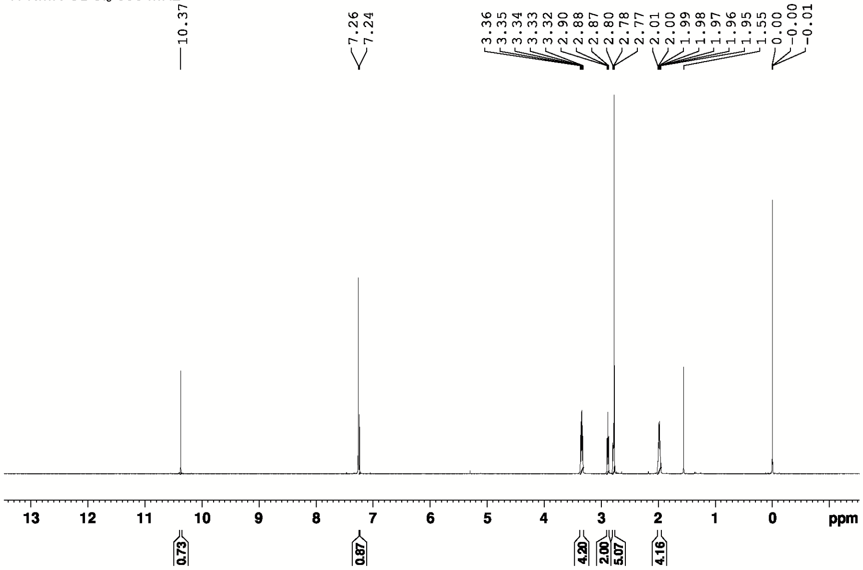

^{13}C-NMR $CDCl_3$ 126 MHz

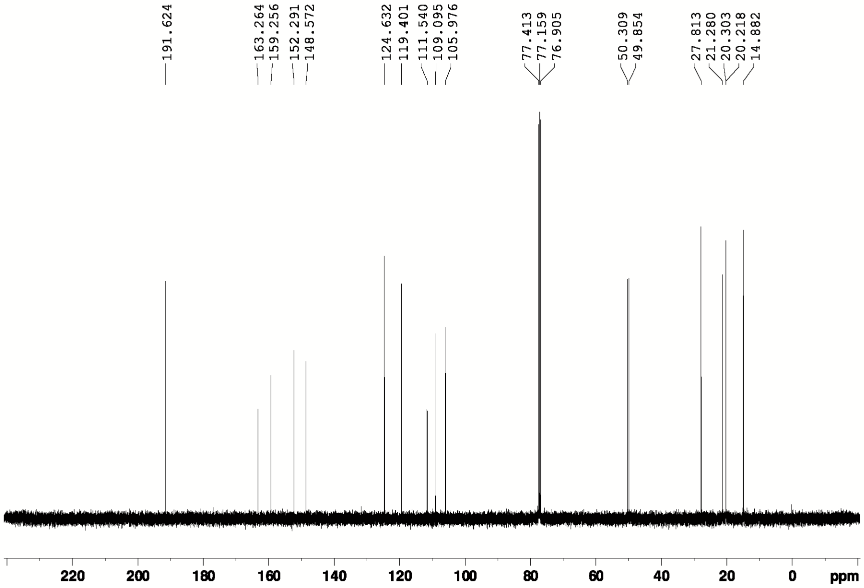

^{1}H NMR and $^{13-}$NMR of 3.

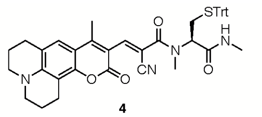

^{1}H-NMR $(CD_3)_2CO$ 500 MHz

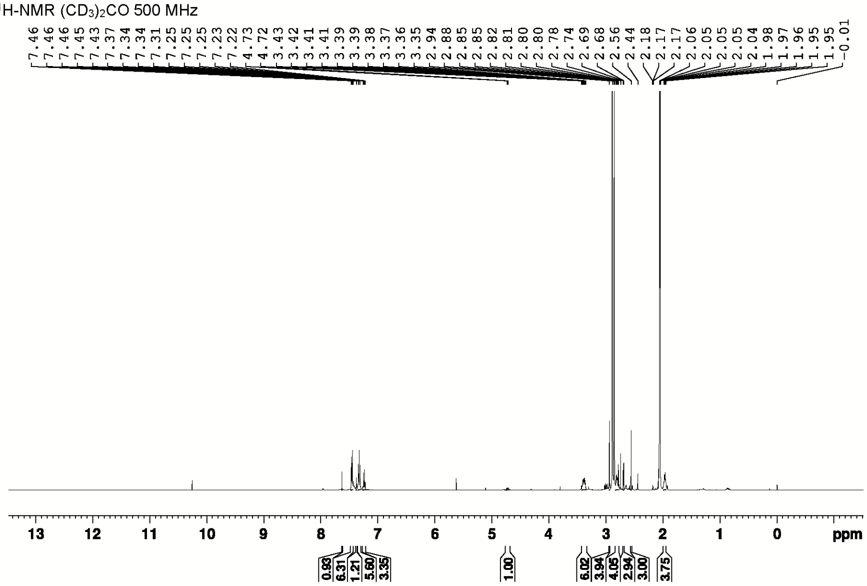

^{13}C-NMR $CDCl_3$ 126 MHz

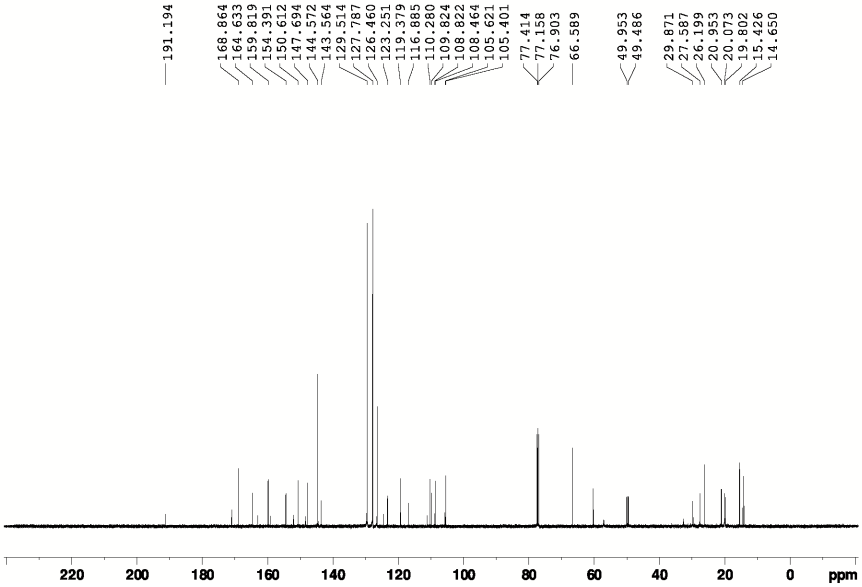

LC trace absorbance at 254 nm

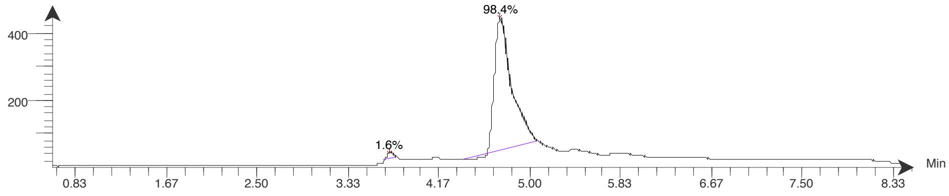

^{1}H-NMR, ^{13}C-NMR, and LC trace of 4.

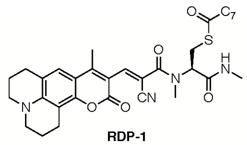

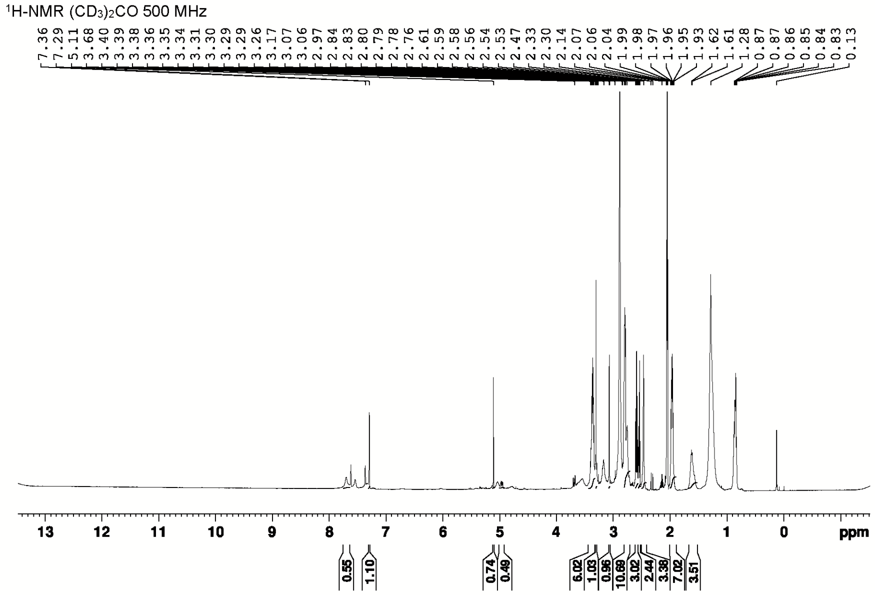

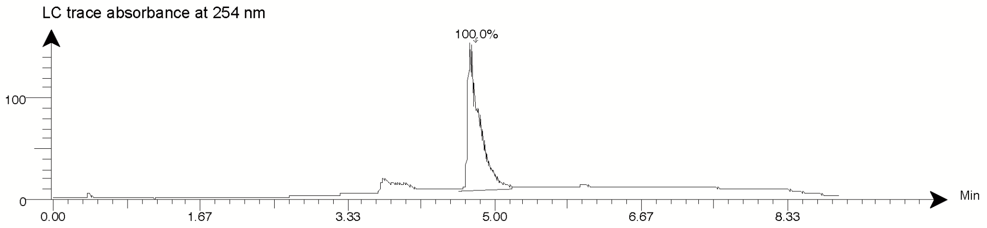

^{1}H-NMR and LC trace of RDP-1.

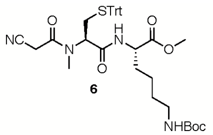

^{1}H-NMR $CDCl_3$ 500 MHz

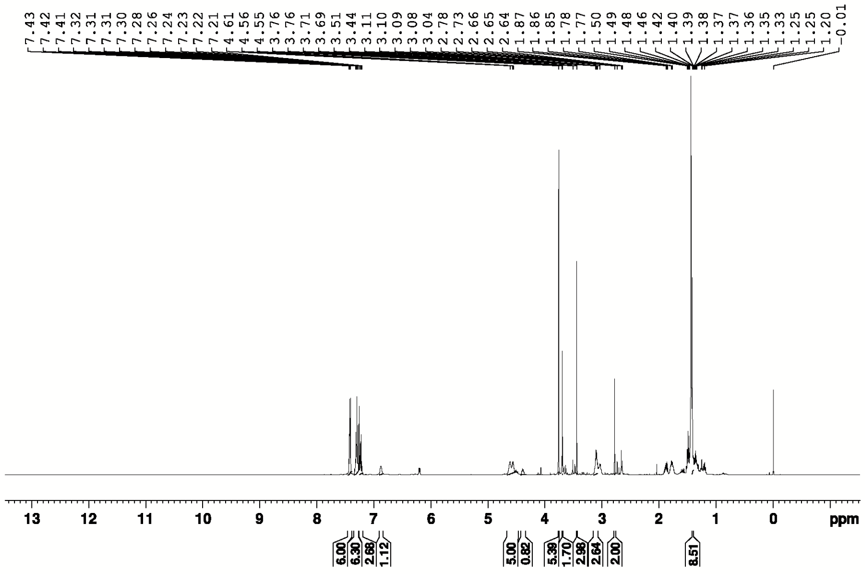

^{13}C-NMR $CDCl_3$ 126 MHz

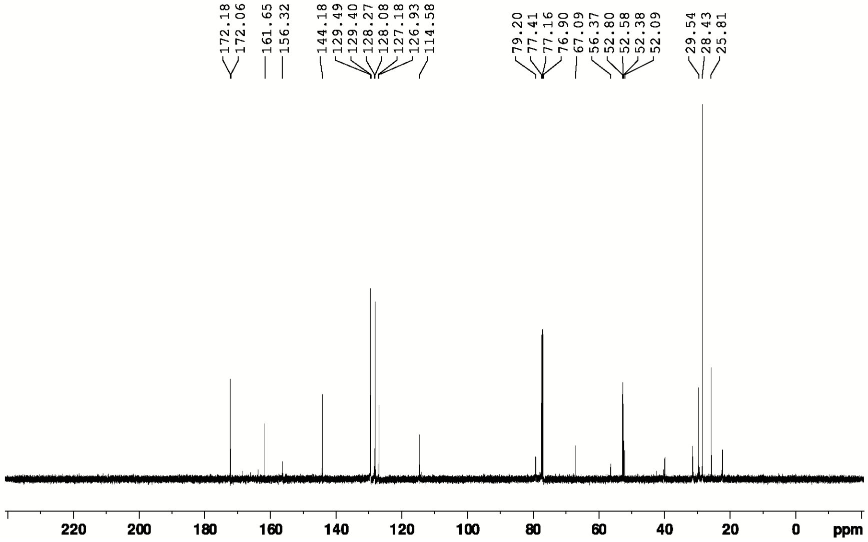

^{1}H-NMR and ^{13}C-NMR of 6.

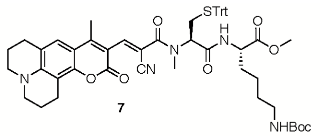

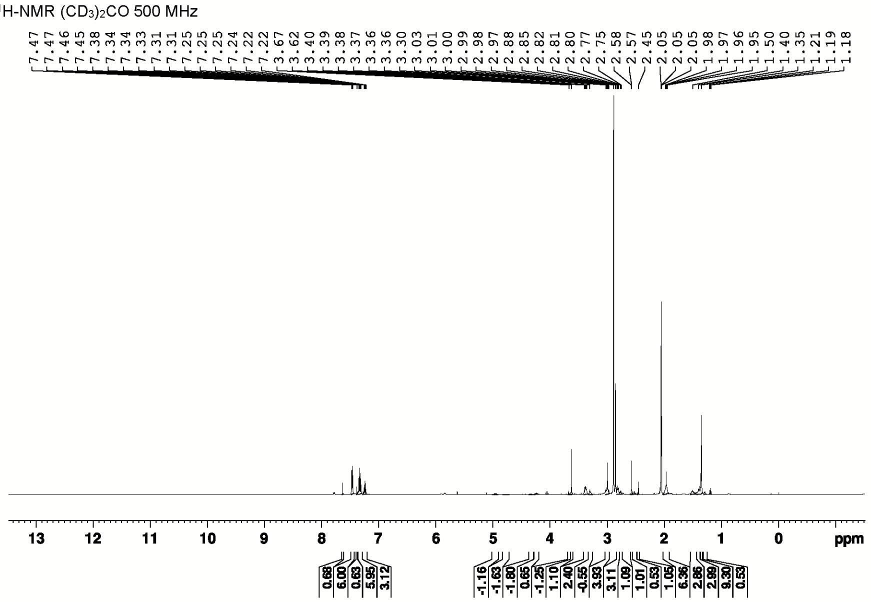

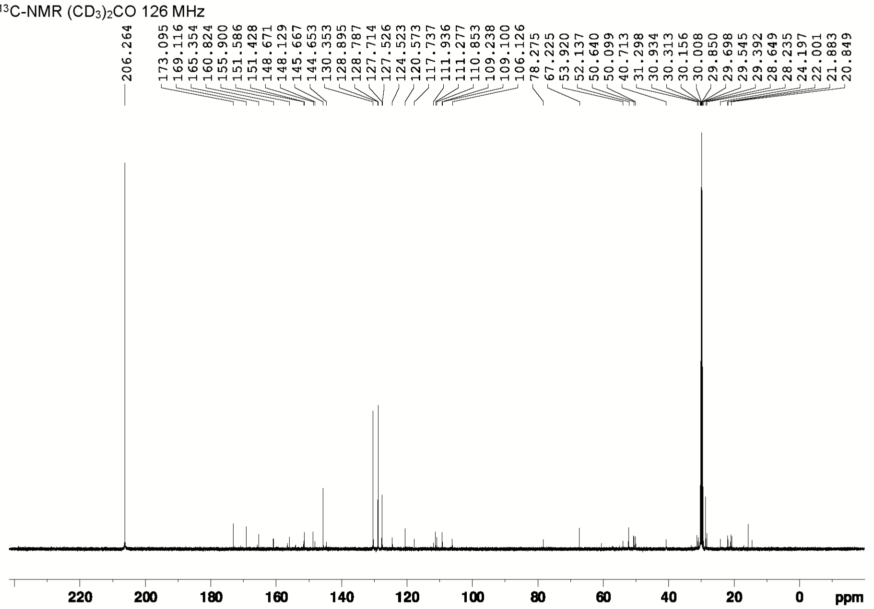

^{1}H-NMR and ^{13}C-NMR of 7.

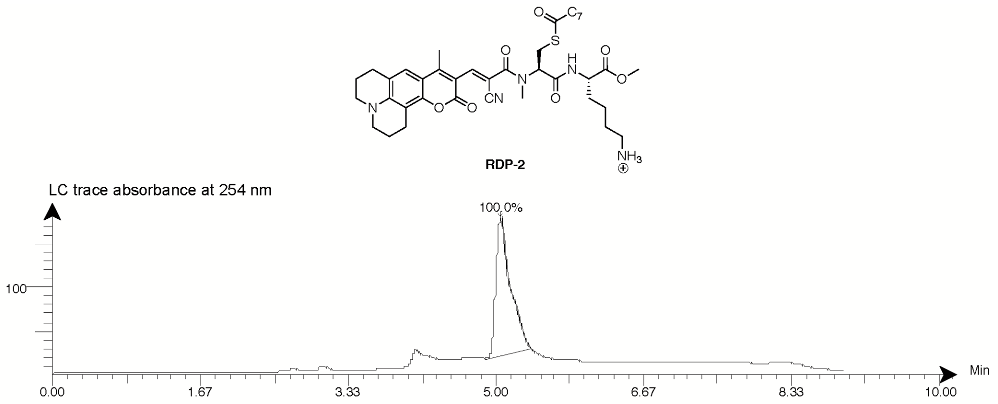

LC trace of RDP-2.
